# Enhancing diagnostic precision in Alzheimer's disease: Impact of comorbidities on blood biomarkers for clinical integration

**DOI:** 10.1002/alz.70931

**Published:** 2025-12-01

**Authors:** Makrina Daniilidou, Ulf Öhlund‐Wistbacka, Göran Hagman, Anna Rosenberg, Nicholas Ashton, Henrik Zetterberg, Kaj Blennow, Anna Matton, Miia Kivipelto

**Affiliations:** ^1^ Division of Clinical Geriatrics Centre for Alzheimer Research Department of Neurobiology Care Sciences and Society Karolinska Institutet Solna Sweden; ^2^ Division of Neurogeriatrics Centre for Alzheimer Research Department of Neurobiology Care Sciences and Society Karolinska Institutet Solna Sweden; ^3^ FINGERS Brain Health Institute Solna Sweden; ^4^ Theme Inflammation and Aging Karolinska University Hospital Solna Sweden; ^5^ Department of Public Health Lifestyles and Living Environments Finnish Institute for Health and Welfare Helsinki Finland; ^6^ Department of Psychiatry and Neurochemistry Institute of Neuroscience and Physiology the Sahlgrenska Academy at the University of Gothenburg Mölndal Sweden; ^7^ Clinical Neurochemistry Laboratory Sahlgrenska University Hospital Mölndal Sweden; ^8^ Department of Neurodegenerative Disease UCL Institute of Neurology London UK; ^9^ UK Dementia Research Institute at UCL London UK; ^10^ Hong Kong Center for Neurodegenerative Diseases Hong Kong China; ^11^ Wisconsin Alzheimer's Disease Research Center University of Wisconsin School of Medicine and Public Health University of Wisconsin–Madison Madison Wisconsin USA; ^12^ Ageing Epidemiology Research Unit (AGE) School of Public Health Faculty of Medicine Imperial College London Charing Cross Hospital London UK; ^13^ Institute of Public Health and Clinical Nutrition University of Eastern Finland Kuopio Finland

**Keywords:** Alzheimer's disease, amyloid beta 42/40 ratio, blood biomarkers, comorbidities, diagnostic accuracy, phosphorylated tau217

## Abstract

**INTRODUCTION:**

Comorbidities may influence Alzheimer's disease (AD) plasma biomarkers. This study aimed to investigate how medical conditions impact AD plasma biomarkers and whether comorbidity‐adjusted models enhance their diagnostic performance.

**METHODS:**

We analyzed key AD plasma biomarkers in 311 memory clinic patients (mean age 59 years, 57% female) at Karolinska University Hospital, Sweden. Biomarkers were measured using single molecular array (SIMOA) and Lumipulse assay. Multivariate linear regressions and receiver operating characteristic/area under the curves (ROC/AUCs) were calculated using clinical diagnosis and cerebrospinal fluid biomarkers as gold standards.

**RESULTS:**

Plasma biomarkers were associated with comorbidities and metabolites such as estimated glomerular filtration rate, homocysteine, and high‐density lipoprotein. Phosphorylated tau (p‐tau)/amyloid beta (Aβ)42 and p‐tau217 had excellent performances in AD pathology classification (AUCs > 0.938). Adjusting for comorbidity measures significantly improved the diagnostic accuracy of Aβ42/40.

**DISCUSSION:**

In conclusion, plasma biomarkers performed robustly despite comorbidity associations, with p‐tau217 emerging as the strongest discriminator. These findings support their potential as diagnostic tools in clinical settings.

**Highlights:**

- Plasma biomarkers for Alzheimer's disease (AD) were assessed in a real‐world memory clinic population.
- Kidney dysfunction and cardiovascular risk factors influenced plasma biomarker levels.
- Phosphorylated tau (p‐tau)217 and p‐tau217/amyloid beta (Aβ)42 ratio showed strongest association with AD pathology.
- Aβ42/40 ratio's accuracy was improved by including comorbidities into the diagnostic algorithms.

## 1 BACKGROUND

Dementia affects millions of individuals worldwide, placing a substantial burden on patients, families, and health‐care systems.^1^ Early and accurate diagnosis is crucial for timely intervention and the development of therapeutic strategies. However, current diagnostic approaches, which rely heavily on clinical evaluation, cerebrospinal fluid (CSF) sampling, and neuroimaging, are often invasive and costly.^2^

Recent advances in the Alzheimer's disease (AD) therapeutics front have led to the approval of the first effective disease‐modifying treatments (DMTs), which are now available for use in many countries.^3^ Lecanemab and donanemab antibodies that clear amyloid plaques from the brain were the first to be approved by US Food and Drug Administration (FDA) and European Medicines Agency (EMA) but there are possibly more to follow.^4^, ^5^ Eligibility for these treatments requires amyloid beta (Aβ) biomarker positivity, creating a need for widely accessible, sensitive, and reliable AD biomarker tests.^6^ As currently available diagnostic assessments (i.e., Aβ/tau positron emission tomography [PET] and CSF biomarkers) are sporadically used due to their invasiveness, cost, and need for specialized equipment, this poses a substantial obstacle to the initiation and monitoring of the novel treatments.^7^

Plasma biomarkers have emerged as a promising alternative tool providing accessibility and cost effectiveness for the diagnosis and monitoring of dementia.^2^, ^8^ Furthermore, they offer the potential for widespread screening in clinical trials and early detection of neurodegenerative processes. Key markers include Aβ peptides, phosphorylated tau (p‐tau) species, neurofilament light chain (NfL) protein, and glial fibrillary acidic protein (GFAP), all of which are involved in the pathophysiological processes of AD and neurodegeneration.^2^, ^7^ Among these, plasma p‐tau217 has shown to highly correlate with both amyloid and tau PET, to predict cognitive status and cognitive decline, and to be in concordance with autopsy‐confirmed amyloid plaques and tau tangles.^9^, ^10^, ^11^, ^12^

Most AD plasma biomarker assays currently available are classified as research use only.^8^ However, the recent FDA approval of a p‐tau217/Aβ42 blood test for diagnostic purposes marks a major advancement toward clinical implementation. Despite this progress, there are still challenges that need to be addressed. Many biomarker assays are still unvalidated for diagnostic use and there are several factors that affect their robustness.^7^ Medical conditions such as chronic kidney disease, hypertension, stroke, myocardial infarction, and increased body mass index (BMI) have been shown to add another layer of complexity in addition to pre‐analytical and analytical factors that are a source of variation on AD plasma biomarker performances.^13^, ^14^, ^15^, ^16^ However, not all biomarkers are as strongly influenced by these conditions. For example, Aβ42/40 and the p‐tau217 to non–p‐tau ratio show a weaker association with kidney function compared to Aβ42 or p‐tau217 concentrations alone.^16^, ^17^ Still, more evidence is needed as most of the data derive from highly selected populations, that is, from clinical trials, or cohorts of healthier individuals at pre‐dementia stages.

In the present study, we aimed to investigate the diagnostic accuracy and clinical utility of key AD‐related plasma biomarkers, including the Aβ42/40 ratio, p‐tau217, ptau217/Aβ42 ratio, p‐tau181, p‐tau231, NfL, and GFAP, with a focus on comorbid conditions, which are frequently found in a real‐world memory clinic population. By leveraging a clinically heterogeneous cohort with individuals at different stages of dementia, we tested these plasma biomarkers in conditions that represent the reality of clinical practice. Specifically, we sought to determine the most significant comorbidity factors associated with the plasma biomarkers as well as to develop models that account for these confounding conditions to enhance the robustness of diagnostic interpretation.

**RESEARCH IN CONTEXT**

1. **Systematic review**: The authors reviewed the literature using traditional sources. The recent development of highly sensitive blood‐based assays for Alzheimer's disease (AD) biomarkers has significantly advanced the dementia field. While a few studies have investigated the influence of comorbidities on blood biomarker levels, evidence from real‐world clinical settings remains limited.
2. **Interpretation**: This study demonstrated that plasma biomarkers are associated with several comorbidities and metabolic factors such as estimated glomerular filtration rate, homocysteine, and high‐density lipoprotein. By incorporating these factors into diagnostic accuracy models, a significant improvement in the performance of the amyloid beta (Aβ)42/40 ratio was observed, while the diagnostic accuracy of phosphorylated tau217/Aβ42 and other biomarkers remained unaffected.
3. **Future directions**: Future studies from larger and more diverse populations are needed for the validation and generalizability of the current findings across different health‐care settings and patient backgrounds.

## 2 METHODS

### 2.1 Study population

The study was conducted at the Karolinska University Hospital Medical Unit Aging Memory clinic in Solna, Stockholm, Sweden. All available information and samples were collected from consecutive patients between April 2018 and April 2021. Τhis specialized outpatient clinic examines individuals with cognitive complaints referred by general practitioners in primary and occupational health care in the catchment area (northern Stockholm), and additionally individuals younger than 70 years in the entire Stockholm region. Most diagnostic examinations are performed within 1 week (fast‐track model). The dementia examination process has been described in detail elsewhere.^18^ Briefly, the harmonized diagnostic evaluation at the memory clinic consists of a comprehensive medical and neurologic examination, medical and informant‐based history, neuropsychological evaluation, blood chemistry, magnetic resonance imaging (MRI), apolipoprotein E (*APOE*) genotyping, and CSF biomarker analysis (e.g., Aβ42, Aβ42/40, p‐tau181, total tau [t‐tau], and NfL). A multidisciplinary team evaluates each patient and sets a consensus diagnosis based on all test results, including biomarkers. Although all participants undergo evaluation within the same fast‐track program, not all assessments were available for every individual. Some examinations (e.g., basic memory tests such as Mini‐Mental State Examination or neuroimaging) are not repeated or documented if performed off site, and MRI or CSF sampling is occasionally omitted due to contraindications or patient unwillingness. BMI is not part of the standardized clinical work‐up and was therefore available only for a subset of participants. The extent of missing data ranged from 45.5% for BMI to < 11% for laboratory tests and CSF biomarkers.

Diagnosis is based on the Diagnostic and Statistical Manual of Mental Disorders Fifth Edition (DSM‐V) criteria, and International Classification of Diseases, Tenth Revision (ICD‐10) coding is used. Patients who do not meet the criteria for mild cognitive impairment (MCI) or dementia are considered to experience subjective cognitive decline (SCD). In this study we considered all consecutive patients with a first diagnostic visit between 2018 and 2021. Of 330 recruited participants, 12 with a diagnosis of “other disease” were excluded, leaving 318 eligible. Plasma biomarker data were not available for 7 individuals, resulting in 311 participants with SCD, MCI, or dementia included in the biomarker analyses (Figure S1 in supporting information).

### 2.2 Comorbidity assessment

The presence of comorbidities was assessed through medical records and current medications, recorded at the baseline visit by licensed medical personnel. Medications were classified according to the Anatomical Therapeutic Chemical (ATC) system, as documented in the medical records. Specifically, agents in categories C01 to C09 were used to define drugs for cardiovascular disorders, C10 for lipid‐lowering agents, A10 for anti‐diabetic treatments, H03A for thyroid disorder medications, and N06A/N05B for antidepressants and anxiolytics. Although these categories encompass pharmacologically heterogeneous agents, this grouping approach was chosen to ensure adequate statistical power and interpretability given the available sample size.

### 2.3 CSF biomarker assessment

CSF samples were collected by lumbar puncture between the L3/L4 or L4/L5 intervertebral space using a 25‐gauge needle. Samples were collected in polypropylene tubes, centrifuged within 2 hours, and analyzed for Aβ42, Aβ40, t‐tau, p‐tau181, and NfL concentrations at Karolinska University Hospital Laboratory in connection to their first diagnostic visit. For samples analyzed until August 21, 2019, biomarkers were measured with commercially available Innotest sandwich enzyme‐linked immunosorbent assays (ELISA; Fujirebio Europe). From August 22, 2019, samples were analyzed with the Lumipulse G‐series (Fujirebio Europe) fully automated chemiluminescent enzyme immunoassay, shown to be highly concordant with the Innotest assay.^19^ Biomarker cutoffs were based on the Karolinska laboratory/manufacturer recommendations. For Innotest, cutoffs were as follows: Aβ42/40 ≤ 0.68, Aβ42 ≤ 550 pg/mL, p‐tau181 ≥ 60 pg/mL, and t‐tau ≥ 400 pg/mL. For Lumipulse, cutoffs were as follows: Aβ42/40 ≤ 0.89, Aβ42 ≤ 599 pg/mL, p‐tau181 ≥ 56.5 pg/mL, and t‐tau ≥ 404 pg/mL. NfL was measured using an NF‐Light ELISA (Catalog no. 10‐7001, Uman Diagnostics). Albumin concentrations in both CSF and serum were measured using the BN ProSpec/Atellica NEPH platform (Siemens Healthineers).

### 2.4 Blood biomarker assessment

Blood was drawn under fasting conditions between 7:00 and 11:00 am. Hemoglobin A1C (HbA1c), total cholesterol, high‐density lipoprotein (HDL), low‐density lipoprotein (LDL), creatinine, homocysteine, sedimentation rate (SR), free thyroxine (free T4), and thyroid‐stimulating hormone (TSH) were analyzed at the certified Karolinska University hospital laboratory. The estimated glomerular filtration rate (eGFR) was calculated from serum creatinine using the Chronic Kidney Disease Epidemiology Collaboration (CKD‐EPI) creatinine‐based equation.^20^

Blood for AD biomarker analysis was collected into sodium heparin tubes (Vacutainer, BD Diagnostics; catalog no. 369623) and then centrifuged at (1500 × g [3000 rpm], +4°C) for 10 minutes. After centrifugation, the samples were aliquoted into polypropylene tubes and stored at −80°C within 30 to 60 minutes of collection. Analysis was conducted at Gothenburg University, Neurochemisty Lab, in a blinded manner with respect to the diagnosis. Levels of plasma Aβ40, Aβ42, GFAP, and NfL were quantified using a multiplexed single molecular array (SIMOA, N4PE; Quanterix). Plasma p‐tau181 and p‐tau231 were assessed using in‐house developed SIMOA assays described elsewhere.^21^, ^22^ Plasma p‐tau217 was analyzed in the Lumipulse fully automated platform G600II using a commercially available kit (Fujirebio Europe).

### 2.5 Neuroimaging assessment

Patients underwent 3T MRI at the clinic (GE Medical systems Discovery MR750 3T) according to a routine protocol comprising T1 fluid‐attenuated inversion recovery (FLAIR), T1 3D gradient recalled echo inversion recovery brain volume imaging (GRE IR BRAVO), T2 FLAIR 3D CUBE, axial T2 PROPELLER 4 mm, axial diffusion‐weighted imaging (DWI) Kaiser score (KS), and Ax SWI 3D KS sequences. Experienced neuroradiologists evaluated the scans and assessed visually medial temporal lobe atrophy (MTA; Scheltens scale, 0–4).^23^ MRI or computed tomography (CT) performed off‐site according to local protocols was considered if MRI was not performed at the clinic.

### 2.6 Statistical analysis

Descriptive statistics including mean, standard deviation (SD), frequency, and percentages were calculated. Differences between categorical variables were investigated by chi‐squared test. Age and education differences between diagnostic, Aβ tau (AT), as well as comorbidity groups were assessed by Kruskal–Wallis test due to non‐normal distributions.

CSF and blood biomarkers were transformed with zero‐skewness logarithmic transformation (Box‐Cox) due to non‐normal distributions and further used in linear/logistic regression and analysis of covariance (ANCOVA) models.

Biomarker differences between diagnostic and AT groups were analyzed by ANCOVAs, adjusting for age and sex. ANCOVA was used for comparisons of AD blood biomarkers between the comorbidity groups, covarying for age, sex, and education, as the groups differed significantly for these factors. Robustness to underlying AD pathology was examined by repeating the ANCOVAs with additional adjustment for CSF Aβ42/40 across all comorbidity categories.

Associations between plasma biomarkers and comorbidity measures were evaluated by linear regression models, in which each AD blood biomarker was set as dependent and each comorbidity measure as independent variable. The models were adjusted for age, sex, education, and diagnosis to evaluate if the associations were independent of these confounding factors.

The discriminatory performances of the AD plasma biomarkers for A+T+ positivity, MCI/all‐cause dementia, AD dementia, and MTA abnormality were assessed by multivariate logistic regressions. A+T+ positivity was determined using the Karolinska University hospital laboratory cutoff values for CSF Aβ42/40 and CSF p‐tau181. MTA abnormality was defined based on age‐adjusted cutoffs published elsewhere.^24^ For the AD versus SCD comparison, the AD group comprised participants with a clinical diagnosis of AD dementia according to DSM‐IV criteria and ICD‐10 code F00 (subtypes F00.0–F00.9). Two models were used for each outcome: (1) a basic model in which each plasma biomarker was included adjusting for age, sex, and education, and (2) a comorbidity‐adjusted model in which the most important comorbidity factors that resulted from the analyses (HDL, homocysteine, eGFR) were added to the variables of the basic model. In addition, basic and comorbidity‐adjusted models were tested for AD plasma biomarker combinations. Area under the receiver operating characteristic curve (AUROC) measures between the basic and comorbidity‐adjusted models were compared with the paired DeLong test (pROC package, R software). For each model we identified the optimal threshold by Youden *J* (sensitivity + specificity − 1). As biomarkers were modeled on a transformed scale, thresholds were back‐transformed to the raw units for reporting.

Regression assumptions were assessed after log transformation of biomarker values. Normality of residuals was evaluated by visual inspection of Q–Q plots and histograms, which did not show major deviations. Linearity and homoscedasticity were examined by plotting standardized residuals against fitted values, in which residuals were approximately randomly scattered around zero without clear patterns. Potential influential outliers were assessed using standardized residuals (> ± 3 SD) and Cook distance. As a sensitivity analysis, all regression models were repeated after excluding potential outliers.

Missing data were handled using listwise deletion in the respective analyses. The number of available cases for each variable is reported in Table 1 and Table S1 in supporting information.

**TABLE 1:** Demographic and clinical characteristics of the study population.

	*N*	
Age, years	311	59.0 (6.6)
Sex, female %	311	57.2
Education, years	289	13.4 (3.2)
SCD/MCI/dementia, %	311	53.7/23.5/23.8
*APOE* ε4, %	305	41.6
MMSE	228	26.0 (3.9)
MoCA	285	22.9 (5.2)
PHQ‐9	276	7.4 (6.0)
Cardiovascular disorder, %	311	50.8
Diabetes, %	311	16.7
Dyslipidemia, %	311	26.0
Depression, %	311	57.6
Thyroid disease, %	311	10
BMI	173	26.3 (4.1)
Systolic BP, mmHg	293	140 (19.1)
Diastolic BP, mmHg	293	86.0 (24.1)
TSH, mE/L	282	2.4 (7.4)
T4 free, pmol/L	282	15.7 (2.5)
Total chol, mmol/L	297	5.3 (1.1)
HDL, mmol/L	297	1.6 (0.5)
LDL, mmol/L	277	3.0 (1.0)
HbA1c, mmol/mol	284	38.3 (8.1)
eGFR, mL/min/1.73 m^2^	296	90.6 (14.3)
Homocysteine, µmol/L	298	12.6 (4.5)
SR, mm/h	292	11.5 (11.5)
CSF/s albumin ratio	279	6.6 (3.3)

*Note*: Continuous data are shown as mean (SD) and categorical as % frequencies.

Abbreviations: *APOE*, apolipoprotein E; BMI, body mass index; BP, blood pressure; CSF/s, cerebrospinal fluid/serum; HbA1c, hemoglobin A1C; HDL, high‐density lipoprotein; LDL, low‐density lipoprotein; MMSE, Mini‐Mental State Examination; MCI, mild cognitive impairment; MoCA, Montreal Cognitive Assessment; PHQ‐9, Patient Health Questionnaire 9; SCD, subjective cognitive decline; SR, sedimentation rate; T4 free, thyroxine; TSH, thyroid‐stimulating hormone.

Unadjusted *P* values are presented in the main text together with effect sizes. In sensitivity analysis, we controlled the false discovery rate (FDR) using the Benjamini–Hochberg (BH) procedure (*q* = 0.05) within families of related tests: (1) regressions—per biomarker across all predictors; (2) ANCOVAs—per comorbidity factor across biomarkers; and (3) AUC comparisons—per classification outcome across biomarkers.

Statistical significance was set to *P* < 0.05. Analyses were performed using SPSS Statistics, version 28.0 (IBM Corp) and R software. Figures were built using R software. Stata software, version 14 (StataCorp) was used for the zero‐skewness logarithmic transformations.

## 3 RESULTS

### 3.1 Study population characteristics

A total of 311 memory clinic patients were included in the study. The mean age (SD) of the participants was 59.0 (6.6) years, 57.2% were female, and 41.6% had at least one *APOE* ε4 allele (Table 1). There were 167 SCD (53.7%), 73 (23.5%) MCI, and 71 (22.8%) dementia patients. AD was the most frequent dementia diagnosis (*N* = 49).

Frequencies of common comorbidities in the total population are presented in Table 1 and Figure S2 in supporting information. More than half of the participants had a cardiovascular condition (50.8%) or depression/anxiety (57.6%). Regarding overall comorbidity burden, 17.7% of the cohort had no comorbidities under investigation, 35% had one condition only, 23.2% had two conditions, and the remainder had three or more. The most frequent combination was cardiovascular disorders and depression/anxiety (Figure S2).

Participants with MCI/dementia were older, less educated, with an overrepresentation of male sex, and a higher frequency of the *APOE* ε4 allele compared to SCD individuals (Table S1). CSF Aβ42/40 and Aβ42 levels were lower in those with MCI/dementia, whereas p‐tau181, t‐tau, and NfL were higher (Table S1). Similarly, plasma Aβ42/40 and Aβ42 were decreased and p‐tau species, ptau217/Aβ42 ratio, NfL, and GFAP were increased in cognitively impaired patients (Table S1). Similar findings were observed comparing A+T+ participants to the rest (Table S1).

### 3.2 AD plasma biomarker differences by comorbidities

To study the impact of comorbidities, we first compared AD plasma biomarker levels in groups with or without common medical conditions (Table 2 and Figures S3–S7 in supporting information). The groups were not balanced in terms of age, sex, and education (Table S2 in supporting information); therefore, all analyses were adjusted for these factors. P‐tau217 and p‐tau217/Aβ42 were decreased in individuals with cardiovascular conditions (*P* = 0.022 and *P* = 0.006) and in those with depression/anxiety (*P* = 0.014 and *P* = 0.039, respectively). Aβ42 and Aβ40 levels were elevated in persons with dyslipidemia (*P* = 0.032 and *P* = 0.003, respectively; Table 2).

**TABLE 2:** Comparisons of mean group differences on AD plasma biomarkers by comorbidities.

Comorbidity	No (mean, SD)	Yes (mean, SD)	*P* value	^a^ *P* value CSF adjusted
Cardiovascular	*N* = 153	*N* = 158		
Aβ42/40 ratio	0.07 (0.02)	0.07 (0.01)	0.920	0.628
Aβ42, ng/L	8.0 (1.9)	8.3 (2.1)	0.178	0.530
Aβ40, ng/L	115 (20.6)	123 (23.5)	0.157	0.243
p‐tau217, ng/L	0.33 (0.56)	0.25 (0.39)	0.022	0.578
p‐tau217/Aβ42 ratio	0.05 (0.09)	0.03 (0.06)	0.006	0.264
p‐tau181, ng/L	9.0 (7.1)	9.5 (6.8)	0.181	0.552
p‐tau231, ng/L	17.6 (5.2)	18.6 (7.5)	0.257	0.917
NfL, ng/L	20.6 (14.9)	21.8 (15.9)	0.529	0.669
GFAP, ng/L	118 (85.3)	123 (76.9)	0.161	0.829
Diabetes	*N* = 259	*N* = 52		
Aβ42/40 ratio	0.07 (0.02)	0.06 (0.01)	0.475	0.013
Aβ42, ng/L	8.1 (1.9)	8.3 (2.3)	0.483	0.902
Aβ40, ng/L	117 (19.5)	127 (32.5)	0.109	0.027
p‐tau217, ng/L	0.30 (0.47)	0.27 (0.53)	0.211	0.849
p‐tau217/Aβ42 ratio	0.04 (0.07)	0.04 (0.08)	0.147	0.895
p‐tau181, ng/L	9.2 (7.1)	9.3 (6.3)	0.180	0.598
p‐tau231, pg/mL	17.9 (5.5)	19.2 (10.2)	0.591	0.176
NfL, ng/L	20.7 (13.5)	23.6 (21.8)	0.562	0.370
GFAP, ng/L	121 (81.5)	120 (79.5)	0.303	0.262
Dyslipidemia	*N* = 230	*N* = 81		
Aβ42/40 ratio	0.07 (0.01)	0.07 (0.02)	0.995	0.855
Aβ42, ng/L	8.0 (1.9)	8.5 (2.2)	0.032	0.105
Aβ40, ng/L	116 (21.3)	128 (23.7)	0.003	0.022
p‐tau217, ng/L	0.29 (0.48)	0.29 (0.48)	0.550	0.522
p‐tau217/Aβ42 ratio	0.04 (0.08)	0.04 (0.07)	0.180	0.857
p‐tau181, ng/L	9.1 (7.4)	9.6 (5.8)	0.505	0.946
p‐tau231, ng/L	17.6 (5.4)	19.6 (8.7)	0.630	0.221
NfL, ng/L	20.6 (13.6)	23.0 (18.8)	0.899	0.447
GFAP, ng/L	119 (82.3)	125 (77.7)	0.877	0.625
Depression	*N* = 132	*N* = 179		
Aβ42/40 ratio	0.07 (0.01)	0.07 (0.01)	0.976	0.632
Aβ42, ng/L	8.1 (2.1)	8.2 (2.0)	0.943	0.901
Aβ40, ng/L	118 (23.4)	120 (21.8)	0.954	0.720
p‐tau217, ng/L	0.36 (0.53)	0.24 (0.34)	0.014	0.114
p‐tau217/Aβ42 ratio	0.05 (0.09)	0.03 (0.05)	0.039	0.220
p‐tau181, ng/L	9.6 (7.6)	9.0 (6.5)	0.302	0.843
p‐tau231, ng/L	18.9 (7.4)	17.6 (5.7)	0.104	0.637
NfL, ng/L	21.3 (15.9)	21.1 (14.6)	0.744	0.975
GFAP, ng/L	121 (73.0)	120 (86.7)	0.100	0.244
Thyroid disease	*N* = 280	*N* = 31		
Aβ42/40 ratio	0.07 (0.02)	0.07 (0.01)	0.340	0.520
Aβ42, ng/L	8.1 (2.0)	8.4 (1.8)	0.961	0.531
Aβ40, ng/L	119 (21.8)	124 (20.7)	0.320	0.188
p‐tau217, ng/L	0.29 (0.49)	0.29 (0.34)	0.435	0.777
p‐tau217/Aβ42 ratio	0.04 (0.08)	0.04 (0.04)	0.531	0.521
p‐tau181, ng/L	9.01 (6.1)	10.8 (12.4)	0.255	0.675
p‐tau231, ng/L	18.1 (6.5)	18.9 (6.1)	0.267	0.608
NfL, ng/L	21.3 (15.4)	20.5 (13.3)	0.758	0.304
GFAP, ng/L	120 (81.4)	124 (79.0)	0.481	0.757

Abbreviations: Aβ, amyloid beta; AD, Alzheimer's disease; CSF, cerebrospinal fluid; GFAP, glial fibrillary acidic protein; NfL, neurofilament light protein; p‐tau, phosphorylated tau; SD, standard deviation.

Notes: Plasma biomarker levels are shown as unadjusted mean (SD). *P* values are calculated from one‐way analysis of covariance adjusted for age, sex and education. “*p*‐value CSF adj” indicates additional adjustment for CSF Aβ42/40 levels. *P* < 0.05 was considered statistically significant.

^a^ Data available for 285 participants.

To assess whether the observed differences were driven by underlying AD pathology, we repeated the models with additional adjustment for CSF Aβ42/40 ratio levels (Table 2). After CSF adjustment, the previously observed differences in the depression group for p‐tau217 and p‐tau217/Aβ42 were no longer significant. Plasma Aβ40 remained significantly lower in the dyslipidemia group (*P* = 0.022), whereas the differences in Aβ42 disappeared. CSF adjustment revealed two additional associations: lower plasma Aβ42/40 (*P* = 0.013) and Aβ40 (*P* = 0.027) in the diabetes group. In sensitivity analyses with FDR correction, the findings became non‐significant (Table S3 in supporting information).

### 3.3 AD plasma biomarker associations with comorbidity measures

**FIGURE 1:**
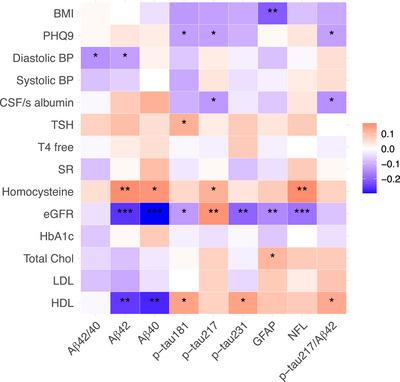
Associations heatmap between comorbidity measures and plasma biomarkers. Standardized beta coefficients from linear regression models adjusted for age, sex, education, and diagnosis indicated in red/blue depending on the direction of the association (red: positive association, blue: negative association). ns *P* ≥ 0.05, *P < 0.05, **P < 0.01, ***P < 0.001. Aβ, amyloid beta; BMI, body mass index; BP, blood pressure; CSF/s, cerebrospinal fluid/serum; eGFR, estimated glomerular filtration rate; GFAP, glial fibrillary acidic protein; HbA1c, hemoglobin A1C; HDL, high‐density lipoprotein; LDL, low‐density lipoprotein; NfL, neurofilament light chain; PHQ‐9, Patient Health Questionnaire 9; p‐tau, phosphorylated tau; SR, sedimentation rate; T4 free, thyroxine; TSH, thyroid‐stimulating hormone

We next investigated associations between comorbidity measures and each AD plasma biomarker (Figure 1). The comorbidity measures included cardiovascular and metabolic health (systolic and diastolic blood pressure, total cholesterol, LDL, HDL, HbA1c, and BMI), kidney function (eGFR), thyroid function (TSH, T4 free), cerebrovascular function (CSF/serum albumin, homocysteine), inflammation (SR), and depressive symptoms (Patient Health Questionnaire 9 [PHQ‐9]). All analyses were adjusted for age, sex, education, and clinical diagnosis. Diagnosis was added as a covariate to rule out the possibility that the relationships found were due to the presence of a neurocognitive disorder. Lower eGFR was significantly associated with increased concentrations of all plasma biomarkers except for p‐tau217 for which the association was positive. Aβ42/40 and p‐tau217/Aβ42 ratios were not associated with eGFR. Homocysteine was associated with higher Aβ42, Aβ40, p‐tau217, and NfL. HDL was associated with lower Aβ42 and Aβ40 and higher p‐tau217/Aβ42, p‐tau181, and p‐tau231. Diastolic blood pressure was related to decreased Aβ42/40 ratio and Aβ42. Total cholesterol was associated with higher GFAP levels. TSH was related to increased p‐tau181. PHQ‐9 scale was associated with lower p‐tau181, p‐tau217, and p‐tau217/Aβ42 ratio. The CSF/serum albumin ratio was related to lower p‐tau217 and p‐tau217/Aβ42 ratio.

The relationship of the plasma biomarkers with BMI was investigated in a subsample of the cohort (*N* = 167), with available BMI data. BMI was found to be associated with decreased levels of GFAP.

Overall, the main comorbidity variables related to the AD plasma biomarkers were eGFR, homocysteine, and HDL. Sensitivity analyses showed that excluding potential outliers did not materially change the results (Figure S8 in supporting information). After multiple testing correction, the overall pattern of associations was sustained, with HDL, homocysteine, and eGFR remaining the most robust predictors (Table S4 in supporting information).

### 3.4 Impact of comorbidities on plasma biomarker accuracies for AD pathology, clinical diagnosis, and MTA detection

We assessed whether accounting for the main comorbidity factors influenced the plasma biomarkers accuracy to discriminate individuals with AD‐specific pathology (A+T+) from the rest (A–T–, A+T–, A–T+), as well as SCD versus MCI/all‐cause dementia (Tables 3, 4 and Tables S5–S6 in supporting information). In the basic models (age, sex, and education adjusted), p‐tau217 and p‐tau217/Aβ42 ratio had the best accuracies in discriminating A+T+ (area under the curve [AUC]: 0.938 and 0.941, respectively) with optimal thresholds of 0.182 pg/mL and 0.024, yielding sensitivities and specificities > 86% (Table S5). A combination of GFAP, NfL, Aβ42/40, and p‐tau achieved excellent performances with AUCs 0.922 to 0.942 (Table 3).

**TABLE 3:** Discrimination performance of plasma biomarker models on A+T+ positivity.

Plasma biomarker	Basic model AUC (95% CI)	Comorbidity‐adjusted model AUC (95% CI)	*P* value
Discrimination of A+T+ positivity			
p‐tau217/Aβ42 ratio	0.941 (0.909–0.973)	0.944 (0.915–0.973)	0.579
p‐tau217	0.938 (0.902–0.973)	0.943 (0.912–0.974)	0.336
GFAP	0.894 (0.849–0.940)	0.907 (0.867–0.946)	0.157
p‐tau231	0.866 (0.813–0.919)	0.877 (0.824–0.930)	0.325
p‐tau181	0.863 (0.813–0.913)	0.874 (0.825–0.923)	0.305
Aβ42/40 ratio	0.792 (0.730–0.854)	0.832 (0.776–0.889)	0.040
Aβ42	0.780 (0.717–0.843)	0.817 (0.759–0.875)	0.0743
NfL	0.776 (0.713–0.840)	0.784 (0.720–0.848)	0.727
Aβ40	0.674 (0.593–0.755)	0.733 (0.662–0.805)	0.113
Aβ42/40, p‐tau217, NfL, and GFAP	0.942 (0.907–0.978)	0.948 (0.918–0.978)	0.300
Aβ42/40, p‐tau181, NfL, and GFAP	0.922 (0.885–0.959)	0.928 (0.894–0.962)	0.115
Aβ42/40, p‐tau231, NfL, and GFAP	0.922 (0.887–0.957)	0.930 (0.898–0.962)	0.083

Abbreviations: Aβ, amyloid beta; AD, Alzheimer's disease; AUC, area under the curve; CI, confidence interval; CSF, cerebrospinal fluid; GFAP, glial fibrillary acidic protein; NfL, neurofilament light protein; p‐tau, phosphorylated tau.

**TABLE 4:** Discrimination performance of plasma biomarker models on MCI/All cause dementia.

Plasma biomarker	Basic model AUC (95% CI)	Comorbidity‐adjusted model AUC (95% CI)	*P* value
Discrimination of MCI/all‐cause dementia			
p‐tau217	0.824 (0.773–0.875)	0.826 (0.776–0.877)	0.572
p‐tau217/Aβ42 ratio	0.821 (0.770–0.871)	0.823 (0.773–0.873)	0.485
NfL	0.805 (0.754–0.857)	0.809 (0.759–0.860)	0.443
GFAP	0.804 (0.751–0.857)	0.807 (0.754–0.859)	0.635
p‐tau231	0.775 (0.721–0.830)	0.781 (0.727–0.835)	0.296
p‐tau181	0.764 (0.708–0.819)	0.769 (0.714–0.824)	0.323
Aβ42/40 ratio	0.745 (0.688–0.803)	0.750 (0.694–0.807)	0.440
Aβ42	0.742 (0.685–0.799)	0.747 (0.690–0.804)	0.529
Aβ40	0.726 (0.667–0.785)	0.732 (0.674–0.790)	0.482
Aβ42/40, p‐tau217, NfL, and GFAP	0.836 (0.786–0.885)	0.841 (0.792–0.890)	0.311
Aβ42/40, p‐tau181, NfL, and GFAP	0.830 (0.781–0.880)	0.837 (0.788–0.886)	0.267
Aβ42/40, p‐tau231, NfL, and GFAP	0.831 (0.782–0.881)	0.836 (0.787–0.885)	0.374

Abbreviations: Aβ, amyloid beta; AUC, area under the curve; CI, confidence interval; GFAP, glial fibrillary acidic protein; MCI, mild cognitive impairment; NfL, neurofilament light protein; p‐tau, phosphorylated tau.

P‐tau217 and p‐tau217/Aβ42 ratio had very good accuracies in discriminating SCD versus MCI/all cause dementia (AUCs: 0.824 and 0.821, respectively; Table 4). Optimal thresholds were 0.197 pg/mL for p‐tau217 and 0.023 for p‐tau217/Aβ42, yielding high specificities but modest sensitivities (49.2%–52.4%; Table S6). Four marker combinations showed again better accuracies (AUCs: 0.830–0.836).

In comorbidity‐adjusted models, the performance for Aβ42/40 ratio in discriminating A+T+ participants was significantly improved (*P* = 0.040; Table 3). The performances for the rest of the models were all improved, although minimally, as they did not differ significantly from their respective basic models. In some cases thresholds and sensitivity–specificity trade‐offs shifted modestly. For example, the threshold for p‐tau217 in discriminating MCI/all‐cause dementia decreased from 0.197 ng/L (sensitivity 49.2%, specificity 89.8%) in the basic model to 0.153 ng/L (sensitivity 55.6%, specificity 83.7%) in the comorbidity‐adjusted model, despite similar AUCs (Table S6).

In a final step, we evaluated plasma biomarker performance in discriminating MTA abnormality and AD from SCD individuals. The AUCs for AD versus SCD were excellent for p‐tau217/Aβ42, all p‐tau isoforms, GFAP, and NfL, as well as for biomarker combinations (AUCs > 0.916; Table S7 in supporting information). Inclusion of comorbidities had no impact on the models, although a trend for improved classification was observed for Aβ42/40 ratio and Aβ42 (*P* < 0.1 for both). For p‐tau217/Aβ42, the optimal threshold was 0.040 (sensitivity 82.5% and specificity 95.2%), which shifted to 0.026 (89.1% sensitivity and 88.4% specificity) after comorbidity adjustment (Table S8 in supporting information). P‐tau217 thresholds were 0.215 pg/mL in the basic and 0.198 pg/mL in the comorbidity‐adjusted model, both yielding sensitivities and specificities > 84% (Table S8).

In contrast, MTA abnormality was detected only modestly (AUCs 0.615–0.705), and comorbidity adjustment did not alter performance (Table S9 in supporting information); accordingly, no clinically useful cut‐offs could be derived for this outcome.

After FDR correction within each outcome, no between‐model AUC differences were significant.

## 4 DISCUSSION

In this study, we evaluated the impact of comorbidities on AD plasma biomarkers using real‐world data and key AD‐related markers. As blood‐based biomarkers gain clinical relevance, understanding how systemic health affects their levels is crucial for accurate interpretation in memory‐clinic populations, in which comorbid conditions are common. Although our cohort was younger and more highly educated, comorbidity levels were comparable to older, less‐educated cohorts,^16^ allowing investigation of these associations in a younger but similarly affected population.

eGFR, homocysteine, and HDL emerged as key factors associated with several AD plasma biomarkers. eGFR, a proxy to renal function,^20^ was related to nearly all biomarkers except for Aβ42/40 and p‐tau217/Aβ42, suggesting a broad systemic influence of kidney function on biomarker levels. This finding aligns with previous reports showing that impaired kidney function is associated with elevated AD plasma biomarkers, likely due to reduced filtration and clearance.^13^, ^14^, ^15^, ^16^, ^17^, ^25^, ^26^ Strikingly, Mielke et al.^15^ reported that the difference in p‐tau217 and p‐tau181 levels between individuals with and without chronic kidney disease (CKD) was comparable to the difference observed between those with and without elevated brain amyloid in a large population‐based cohort. Similarly, in our study, p‐tau217 levels in CKD participants (eGFR < 60 mL/min/1.73 m^2^, *N* = 7) were comparable to those observed in the overall A+T+ group. Among the CKD‐positive individuals, four were A–T– (mean p‐tau217 = 0.44 ng/L) and three were A+T+ (mean = 0.91 ng/L), whereas the overall A–T– group had a mean of 0.18 ng/L. Although based on a small number of cases, these findings suggest that reduced kidney function could elevate plasma p‐tau217 independently of AD pathology, underscoring the need to consider renal function when interpreting plasma biomarker levels.

Homocysteine, a metabolic byproduct often linked to cerebrovascular damage, was positively correlated with Aβ42, Aβ40, p‐tau217, and NfL. Elevated homocysteine has been widely recognized as a risk factor for both cognitive decline and cerebrovascular disease,^27^, ^28^ yet to our knowledge, there are no previous studies investigating the relation of homocysteine with AD plasma biomarkers. However, it has been shown that cerebrovascular conditions such as stroke and transient ischemic attack increase the levels of Aβ peptides, p‐tau, and NfL.^13^, ^14^, ^15^ In a clinical trial, it was further shown that hypoxic–ischemic injury caused by cardiac arrest, transiently increases and alters the dynamics of p‐tau217 and NfL.^29^ Although the mechanism causing the increase of AD‐related biomarkers in the bloodstream due to cerebrovascular damage is currently unknown, it could be due to blood–brain barrier leakage.

Individuals with dyslipidemia exhibited increased Aβ42 and Aβ40 levels and this finding was sustained for Aβ40 even after adjusting for amyloid pathology, suggesting that these alterations may reflect independent effects of lipid dysmetabolism or its treatment. Lower HDL was associated with higher Aβ42 and Aβ40, as well as with lower p‐tau181, p‐tau231, and p‐tau217/Aβ42 ratio. These findings align with previous research showing that both dyslipidemia and decreased HDL are related to higher Aβ42 and Aβ40 levels in cognitively healthy individuals.^26^ Moreover, lipid‐lowering medication has been associated with increased plasma Aβ42 and Aβ40 in patients across the AD continuum.^16^ Statins have demonstrated some beneficial effects on cognition and AD progression,^30^, ^31^ which could explain the observed Aβ elevations in treated individuals. Nevertheless, it cannot be excluded that lipid metabolism may influence plasma biomarker levels through systemic mechanisms as also indicated by our results.

Participants with cardiovascular conditions exhibited significantly lower p‐tau217 and p‐tau217/Aβ42 ratio; however, these differences disappeared after controlling for CSF Aβ42/40. This indicates that the findings were most likely driven by an associated AD pathology rather than the comorbidity itself. In addition, higher diastolic blood pressure was associated with lower Aβ42 and Aβ42/40. Previous studies have reported mixed associations between cardiovascular conditions and AD plasma biomarkers, most of which were examined in community‐based populations. A recent study^15^ showed that hypertension and myocardial infarction were related to higher p‐tau217, whereas others found instead an increase in Aβ42/40 ratio, Aβ42, and Aβ40.^13^, ^16^, ^26^, ^32^ Interestingly, a study on patients with coronary heart failure showed that the use of the angiotensin receptor blocker sacubitril–valsartan reduced plasma Aβ42/40 ratio by 30%, a higher change compared to the decrease seen in AD patients.^33^ Whether these changes reflect true neuromodulatory effects or are confounded by underlying amyloid pathology needs to be determined in future studies.

Diabetes was associated with lower Aβ42/40 and higher Aβ40 levels after controlling for amyloid pathology, suggesting that diabetes and its pharmacological regulation may modulate plasma biomarker levels through peripheral mechanisms. By contrast, HbA1c, a marker of insulin resistance, was not related to any of the biomarkers in our cohort. Previous studies have shown elevated plasma Aβ42 and Aβ40 in diabetes,^13^, ^16^, ^26^ and that the differences between diabetic and non‐diabetic individuals were sustained after adjusting for brain amyloid pathology, reinforcing that this effect may reflect systemic metabolic regulation rather than central amyloid burden.^13^, ^16^, ^26^ Furthermore, it was shown that these biomarkers were associated with increased HbA1c.^26^

Higher BMI was linked to lower GFAP levels in a sub‐sample of our cohort. The influence of BMI on plasma AD biomarkers has been well documented. In general, increased BMI is shown to be linked to decreased GFAP and NfL, and increased Aβ42 and Aβ40, while p‐tau and Aβ42/40 ratio are minimally affected.^13^, ^14^, ^16^ A plausible explanation of the confounding effect of BMI could be the increased blood volume which confers dilution of the biomarkers in plasma. However, Pichet Binette et al.^16^ showed that the effect of BMI was weakened for all biomarkers except NfL when adjusting for Aβ PET, suggesting that it might not solely be a matter of protein dilution.

Participants with depression or anxiety exhibited lower p‐tau217 and p‐tau217/Aβ42 ratio, and these differences were sustained after controlling for CSF Aβ4240 levels. Depressive symptoms were associated with lower levels of these biomarkers and p‐tau181 in our study. The persistence of these associations after adjusting for AD pathology or clinical diagnosis suggests that the observed effects on plasma p‐tau may reflect independent consequences of depression or psychotropic medication use.

Our study further explored whether adjusting for eGFR, homocysteine, and HDL improves the discriminative accuracy of plasma biomarkers. The results showed that comorbidities had minimal influence on most models, except for a significant improvement in the Aβ42/40 ratio's ability to discriminate A+T+ participants. Our findings align with previous research demonstrating the relative robustness of the Aβ42/40 ratio in predicting AD pathology.^34^, ^35^, ^36^ However, its sensitivity to comorbidity adjustments together with known limitations such as low fold changes between clinical groups^11^ and potential peripheral Aβ production^37^ highlight the necessity of considering certain comorbidity factors when interpreting results.

Among all biomarkers, p‐tau217 and the p‐tau217/Aβ42 ratio had the best performances in discriminating individuals with AD pathology and were the most effective in distinguishing SCD from MCI/all‐cause dementia, confirming previous studies.^36^, ^38^, ^39^, ^40^ Combining GFAP, NfL, Aβ42/40, and p‐tau217 yielded similarly high classification accuracies. While overall discrimination remained stable, we observed small shifts in thresholds and corresponding sensitivity/specificity after comorbidity adjustment. Notably, the p‐tau217 threshold for discriminating MCI/dementia was lower after comorbidity adjustment, illustrating how cut‐offs may change even when overall AUC remains unaffected and that comorbidities can influence clinically relevant operating points.

Notably, plasma p‐tau217 showed higher specificity than sensitivity for detecting MCI/all‐cause dementia. This likely reflects the heterogeneity of these syndromic outcomes and the AD‐specific nature of the biomarker. Thus, it appears more robust for ruling out a neurocognitive disorder in unselected memory populations, as suggested by others.^7^, ^41^ Importantly, its robustness depends on the context of use: when the outcome was defined biologically (A+T+), the assay showed high accuracy, with both sensitivity and specificity > 90%.

A key strength of this study is the deeply phenotyped cohort from a tertiary clinic, incorporating real‐world data and accounting for comorbid conditions. Importantly, we assessed all core AD‐related biomarkers, including p‐tau231, whose association with comorbidities has not been previously explored.

This study has some limitations. Comorbidities were assessed based on medical diagnoses and medication use; however, some drugs may be prescribed for other indications, with variable adherence, duration, and dosage, and preventive prescriptions cannot be excluded. Multimorbidity interactions were not accounted for, which could also influence results. As such, the reported associations reflect total rather than independent effects and overlap between conditions may partially explain some findings. FDR sensitivity analyses confirmed the robustness of key associations but rendered others non‐significant, underscoring the exploratory nature of the results. The relatively young mean age and higher education level of our cohort may limit the generalizability of our findings, as biomarker profiles and cognitive trajectories could differ in older and less‐educated groups. Replication in larger, more representative cohorts is important to confirm generalizability. Finally, our findings are cross‐sectional, limiting conclusions on causality.

## 5 CONCLUSION

In conclusion, plasma biomarkers showed robust performance despite associations with comorbidities, with p‐tau217 emerging as the strongest discriminator of AD‐related classifications. These findings support the potential utility of plasma biomarkers as diagnostic and stratification tools in clinic settings.

## CONFLICT OF INTEREST STATEMENT

Henrik Zetterberg has served on scientific advisory boards and/or as a consultant for AbbVie, Alector, Annexon, Artery Therapeutics, AZTherapies, CogRx, Denali, Eisai, Nervgen, Novo Nordisk, Pinteon Therapeutics, Red Abbey Labs, Passage Bio, Roche, Samumed, Siemens Healthineers, Triplet Therapeutics, and Wave; has given lectures in symposia sponsored by Cellectricon, Fujirebio, Alzecure, Biogen, and Roche; and is a co‐founder of Brain Biomarker Solutions in Gothenburg AB (BBS), which is a part of the GU Ventures Incubator Program (outside submitted work). Miia Kivipelto has served on scientific advisory boards at Eisai, BioArctic, Eli Lilly, Nestlé, and Combinostics. All other authors reported no biomedical financial interests or potential conflicts of interest. Author disclosures are available in the supporting information.

## CONSENT STATEMENT

The Karolinska University Hospital electronic database and biobank for clinical research (GEDOC) and this study have received ethical approval (Regional Ethical Review Board in Sweden; Dnr 2011/1987‐31/4 and 2020‐06484). All patients provided written informed consent. The study was performed in accordance with the ethical standards as described in the 1964 Declaration of Helsinki and its later amendments.

## Supporting information



Supporting Information

Supporting Information
