# Supplementary material for: Enhancing diagnostic precision in Alzheimer's disease: Impact of comorbidities on blood biomarkers for clinical integration
Source: Alzheimers Dement. 2025 Dec 1;21(12):e70931. doi: 10.1002/alz.70931 (PMC12668905; doi:10.1002/alz.70931)
Supplement: Supplementary file 2 — Supporting Information [file ALZ-21-e70931-s001.pdf]

Supplementary material

Figure S1……………………………………………………………………..p. 2

Table S1………………………………………………………………………p. 3

Figure S2…………………………………………………………….……….p. 5

Figures S3-S7………………………………………………………………...p. 6

Table S2………………………………………………………………………p. 11

Table S3………………………………………………………………………p. 13

Figure S8……………………………………………………………………..p. 16

Table S4……………………………………………………………………...p. 18

Table S5……………………………………………………………………...p. 23

Table S6……………………………………………………………………...p. 24

Table S7……………………………………………………………………...p. 25

Table S8……………………………………………………………………...p. 26

Table S9……………………………………………………………………...p. 27

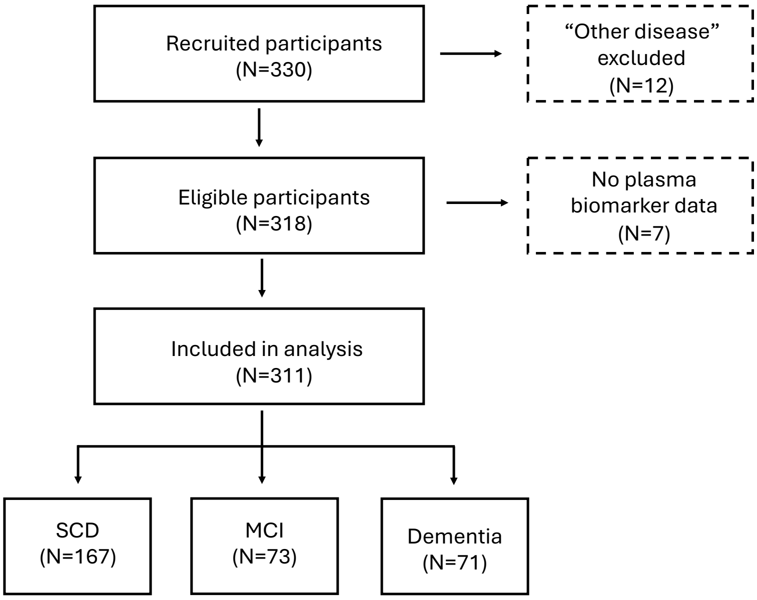

Figure S1. Participant flowchart. Of 330 recruited participants, 12 with a diagnosis of "Other disease" were excluded, leaving 318 eligible. Among these, 311 had plasma biomarker data and were included in the analyses.

Table S1. Demographic and biomarker characteristics of the study population.

	N	Total (N=311)	SCD (N=167)	MCI /Dementia (N=144)	P	A-T-/A-T+/A+/T- (N=220)	A+T+ (N=59)	P
**Age**	311	59.0 (6.6)	57.1 (6.5)	61.3 (6.1)	**<0.001**	58.2 (6.6)	61.8 (5.8)	**<0.001**
**Sex, % female**	311	57.2	64.7	48.6	**0.005**	57.7	59.3	0.641
**Education, years**	289	13.4 (3.2)	14.0 (3.1)	12.6 (3.2)	**<0.001**	13.5 (3.4)	13.0 (2.7)	0.226
**APOE4, %**	305	41.6	33.1	51.8	**0.001**	35.3	63.2	**<0.001**
**CSF biomarkers**								
**Aβ42/40 ratio**	279	0.08 (0.03)	0.09 (0.02)	0.07 (0.03)	**<0.001**	0.09 (0.02)	0.05 (0.01)	**<0.001**
**Aβ42, ng/L**	279	949 (393)	1108 (338)	764 (370)	**<0.001**	1052 (369)	565 (184)	**<0.001**
**p-tau181, ng/L**	280	50.0 (30.2)	39.7 (17.8)	62.4 (36.7)	**<0.001**	37.5 (13.1)	97.5 (29.8)	**<0.001**
**t-tau, ng/L**	280	347 (217)	273 (131)	431 (262)	**<0.001**	268 (132)	640 (223)	**<0.001**
**NfL, ng/L**	279	1070 (1111)	764 (387)	1425 (1506)	**<0.001**	984 (1029)	1388 (1337)	**<0.001**
**Plasma biomarkers**								
**Aβ42/40 ratio**	311	0.07 (0.01)	0.07 (0.01)	0.06 (0.01)	**0.003**	0.07 (0.01)	0.06 (0.01)	**<0.001**
**Aβ42, ng/L**	311	8.2 (2.0)	8.5 (1.9)	7.9 (2.1)	**0.010**	8.5 (2.0)	7.2 (1.7)	**<0.001**
**Aβ40, ng/L**	311	119 (22.0)	117 (20.0)	121 (24.9)	0.954	119 (21.1)	121 (26.6)	0.475
**p-tau217, ng/L**	306	0.13 (0.17)	0.36 (0.65)	0.61 (0.60)	**<0.001**	0.18 (0.38)	0.75 (0.60)	**<0.001**
**p-tau217/ Aβ42 ratio**	306	0.02 (0.02)	0.05 (0.08)	0.10 (0.11)	**<0.001**	0.02 (0.05)	0.12 (0.11)	**<0.001**

**p-tau181, ng/L**	311	9.2 (6.8)	7.5 (4.9)	11.4 (8.4)	**<0.001**	7.9 (4.8)	14.7 (9.9)	**<0.001**
**p-tau231, ng/L**	310	18.1 (6.5)	16.0 (3.9)	20.6 (7.9)	**<0.001**	16.6 (4.3)	24.9 (9.1)	**<0.001**
**NfL, ng/L**	310	21.2 (15.1)	16.5 ‘(9.0)	26.7 (18.7)	**<0.001**	20.1 (15.6)	26.8 (15.0)	**0.002**
**GFAP, ng/L**	310	121 (81)	92.0 (65.6)	154 (84.8)	**<0.001**	103 (73.1)	202 (75.4)	**<0.001**

Data are shown as unadjusted mean (SD), unless otherwise stated. Between-group differences were calculated by Kruskal-Wallis test for age and education. One-way ANCOVA was applied for analysis of CSF and plasma biomarkers, adjusting for age and sex. Chi square was used for categorical data. P<0.05 was considered statistically significant.

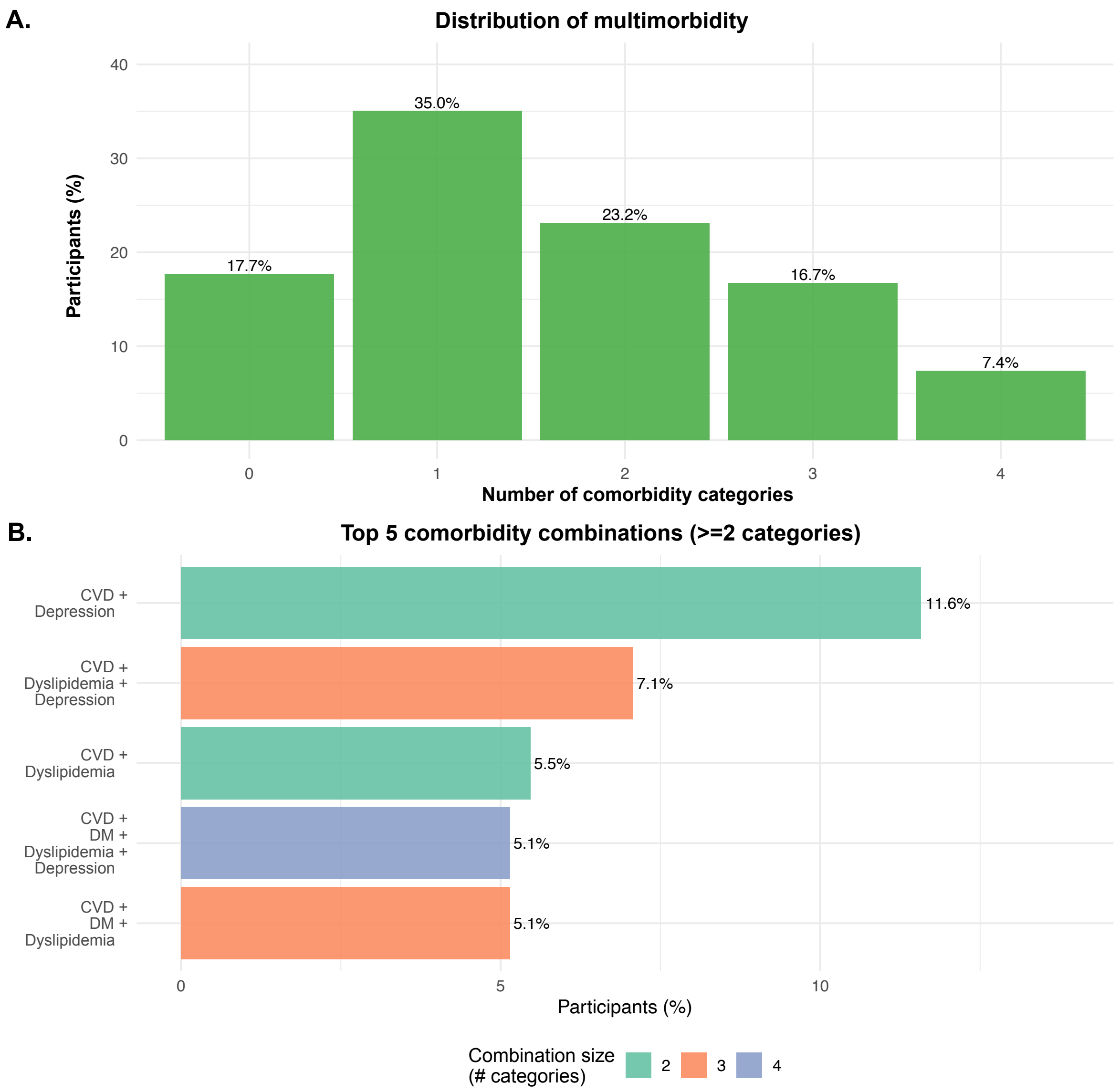

Figure S2. A. % Frequencies of participants by number of comorbidity categories. **B.** Five most frequent combinations of ≥2 comorbidity categories. Abbreviations: CVD, cardiovascular disease; DM, Diabetes Mellitus.

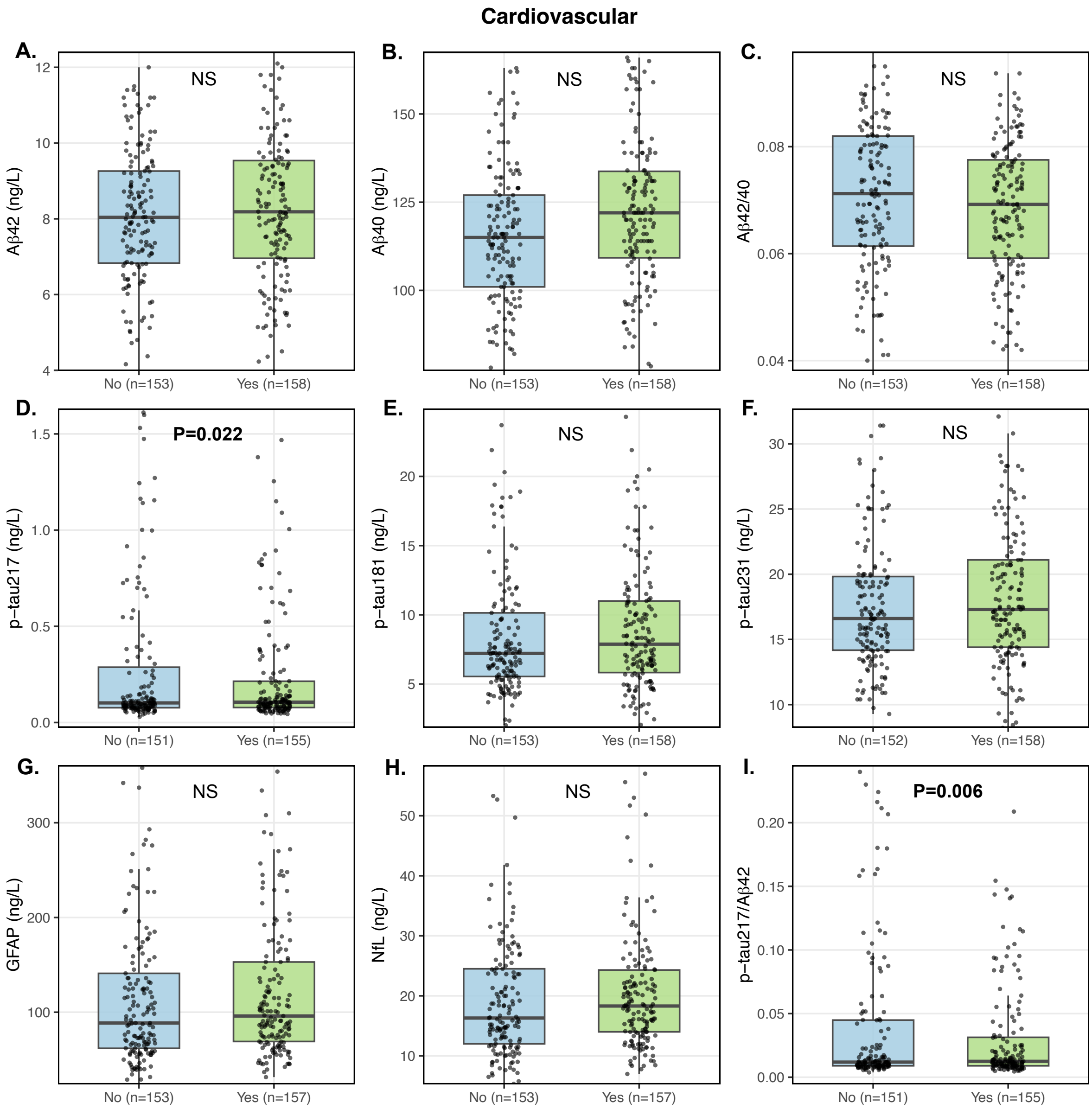

Figure S3. A-I. Boxplots of raw plasma biomarker concentrations stratified by cardiovascular disease groups. Boxes represent the median and interquartile range, with individual values shown as dots. Numbers below each box indicate group size. For visualization, only the central 95% of data are shown (outliers excluded), but all data were included in analyses. P values are from one-way ANCOVA adjusted for age, sex, and education. Abbreviations: Aβ, amyloid β; GFAP, glial fibrillary acidic protein; NfL, neurofilament light protein; NS: statistically non-significant; p-tau, phosphorylated tau.

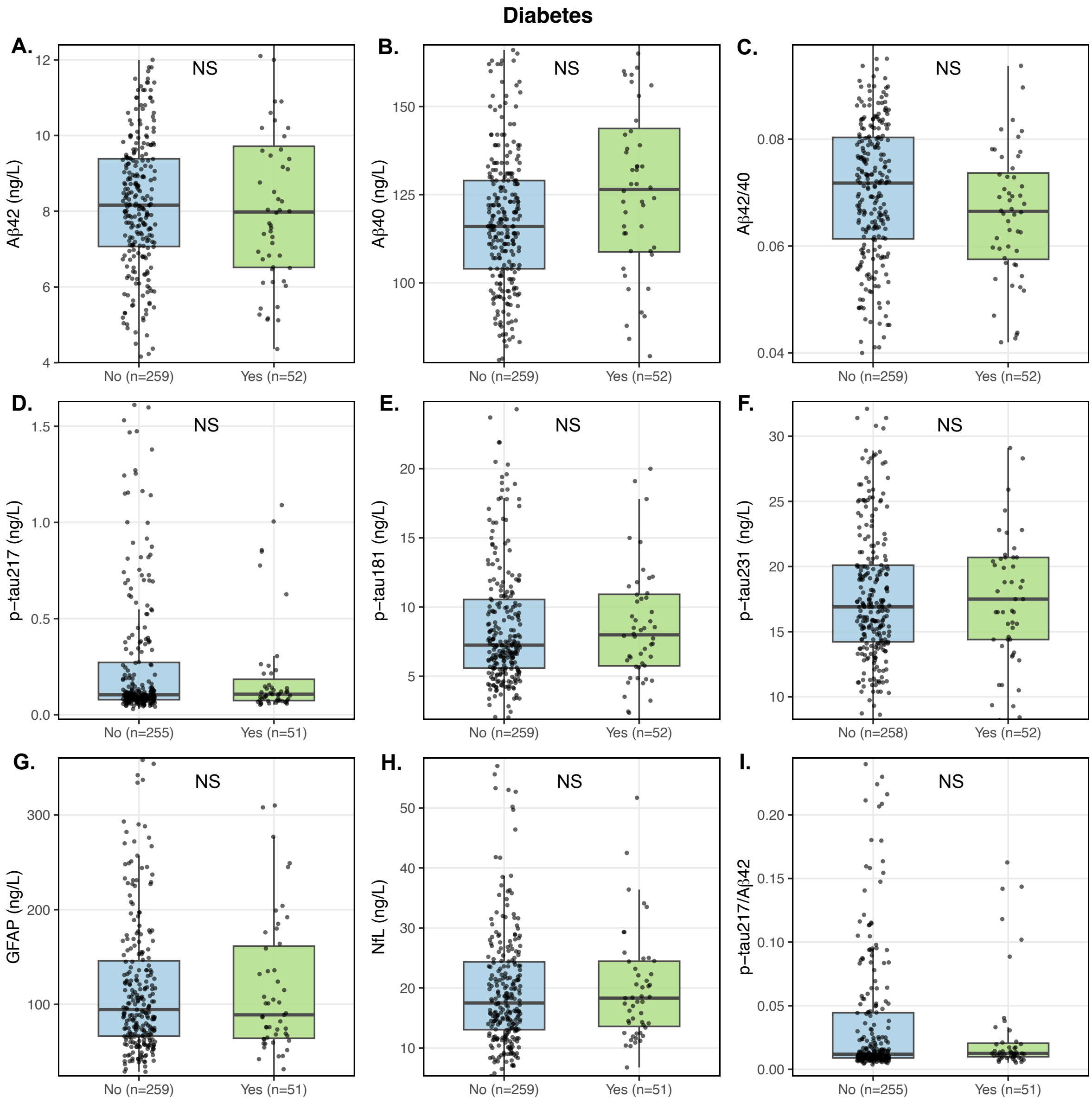

Figure S4. A-I. Boxplots of raw plasma biomarker concentrations stratified by diabetes groups. Boxes represent the median and interquartile range, with individual values shown as dots. Numbers below each box indicate group size. For visualization, only the central 95% of data are shown (outliers excluded), but all data were included in analyses. P values are from one-way ANCOVA adjusted for age, sex, and education. Abbreviations: Aβ, amyloid β; GFAP, glial fibrillary acidic protein; NfL, neurofilament light protein; NS: statistically non-significant; p-tau, phosphorylated tau.

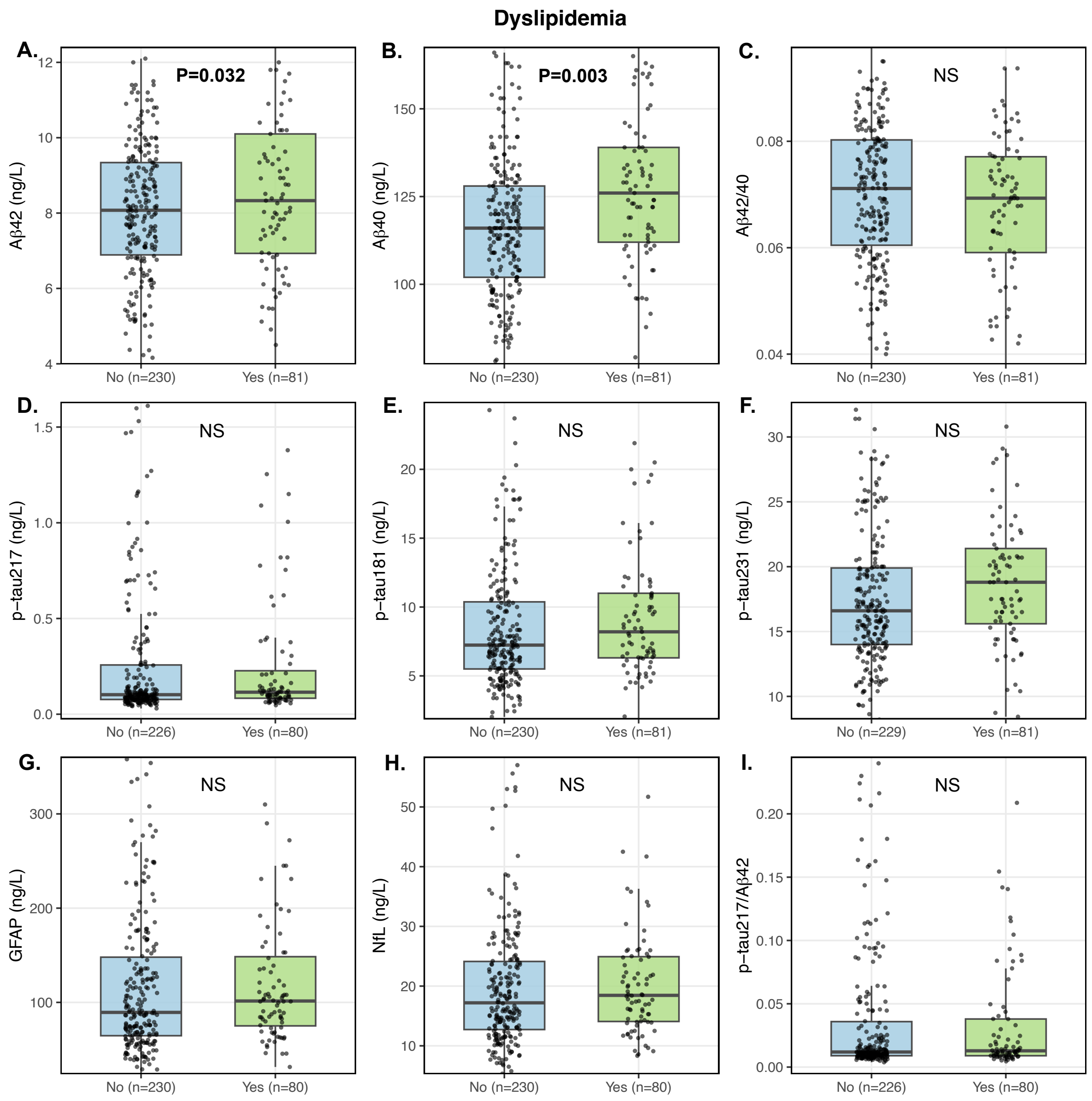

Figure S5. A-I. Boxplots of raw plasma biomarker concentrations stratified by dyslipidemia groups. Boxes represent the median and interquartile range, with individual values shown as dots. Numbers below each box indicate group size. For visualization, only the central 95% of data are shown (outliers excluded), but all data were included in analyses. P values are from one-way ANCOVA adjusted for age, sex, and education. Abbreviations: Aβ, amyloid β; GFAP, glial fibrillary acidic protein; NfL, neurofilament light protein; NS: statistically non-significant; p-tau, phosphorylated tau.

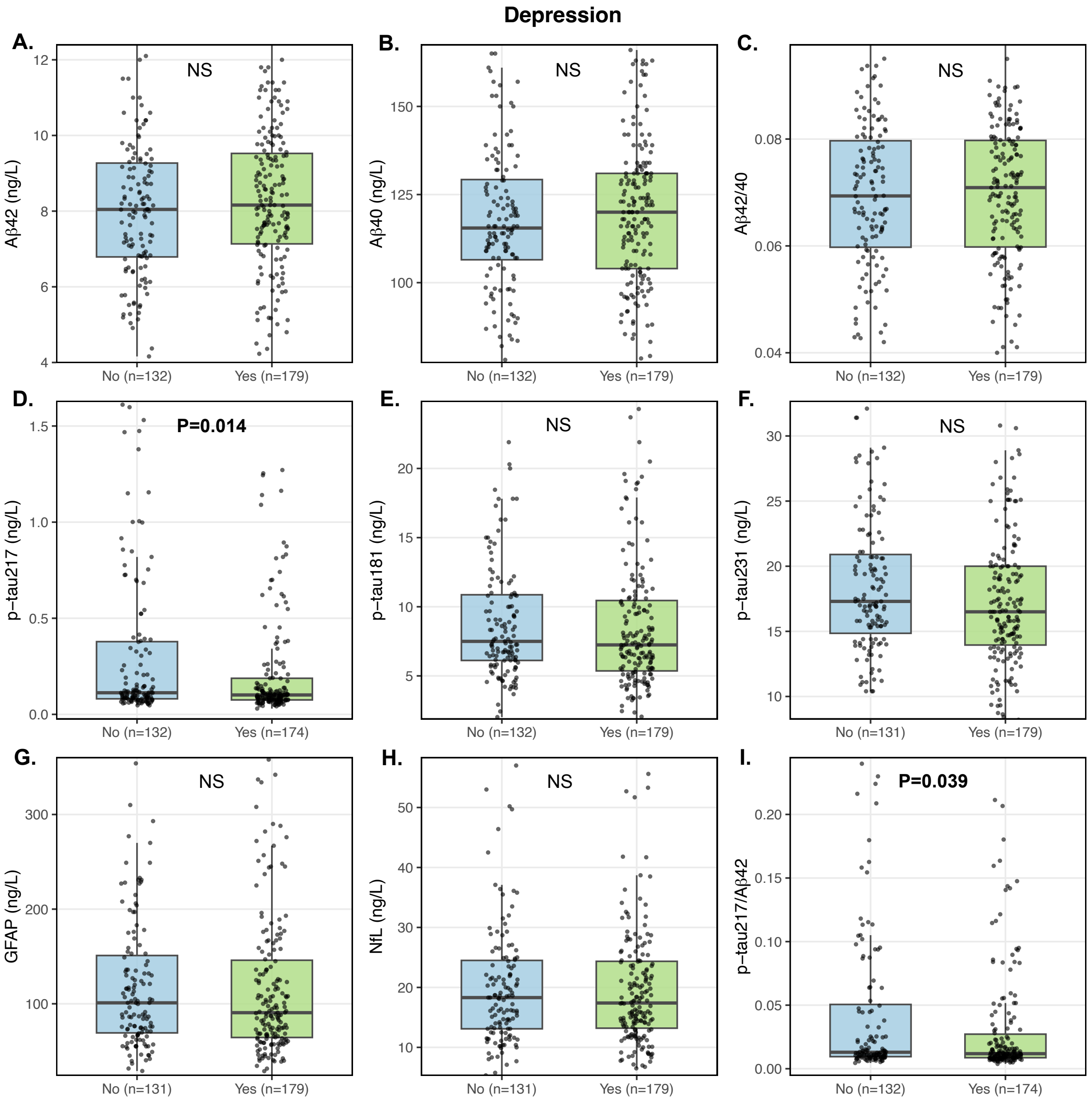

Figure S6. A-I. Boxes represent the median and interquartile range, with individual values shown as dots. Numbers below each box indicate group size. For visualization, only the central 95% of data are shown (outliers excluded), but all data were included in analyses. P values are from one-way ANCOVA adjusted for age, sex, and education. Abbreviations: Aβ, amyloid β; GFAP, glial fibrillary acidic protein; NfL, neurofilament light protein; NS: statistically non-significant; p-tau, phosphorylated tau.

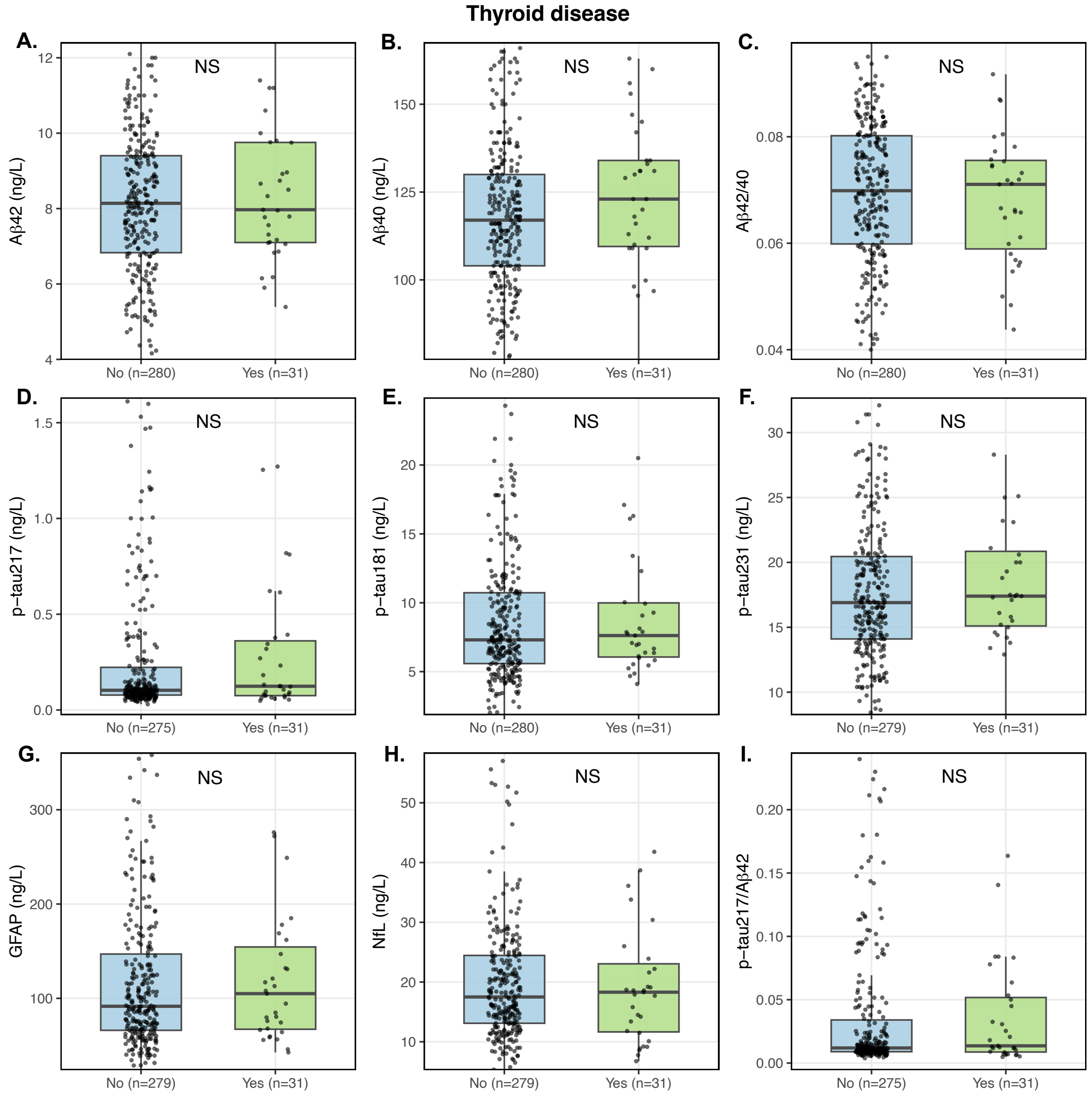

Figure S7. A-I. Boxplots of raw plasma biomarker concentrations stratified by thyroid disease groups. Boxes represent the median and interquartile range, with individual values shown as dots. Numbers below each box indicate group size. For visualization, only the central 95% of data are shown (outliers excluded), but all data were included in analyses. P values are from one-way ANCOVA adjusted for age, sex, and education. Abbreviations: Aβ, amyloid β; GFAP, glial fibrillary acidic protein; NfL, neurofilament light protein; NS: statistically non-significant; p-tau, phosphorylated tau.

Table S2. Comparisons of demographic and clinical characteristics by comorbidities.

Medication	No	Yes	P value
**Cardiovascular**	**N=153**	**N=158**	
Age, mean (SD)	56.7 (7.1)	61.2 (5.3)	**<0.001**
Sex, % female	64.7	50.0	**0.012**
Education, mean (SD)	13.7 (3.1)	13.0 (3.3)	**0.046**
Diagnosis, % SCD/MCI/Dementia	57.5/22.2/20.3	50.0/24.7/25.3	0.389
APOE4, %	45.0	38.3	0.283
[1]CSF Aβ42/40 ratio	0.08 (0.03)	0.08 (0.03)	0.374
**Diabetes**	**N=259**	**N=52**	
Age, mean (SD)	58.5 (6.8)	61.3 (5.0)	**<0.001**
Sex, % female	61.4	36.5	**0.002**
Education, mean (SD)	13.5 (3.1)	12.6 (3.9)	0.108
Diagnosis, % SCD/MCI/Dementia	55.6/23.2/21.2	44.2/25.0/30.8	0.243
APOE4, %	43.7	31.4	0.140
[1]CSF Aβ42/40 ratio	0.08 (0.03)	0.09 (0.03)	0.305
**Dyslipidemia**	**N=230**	**N=81**	
Age, mean (SD)	58.0 (6.9)	61.8 (4.7)	**<0.001**
Sex, % female	62.2	43.2	**0.005**
Education, mean (SD)	13.5 (3.2)	12.9 (3.4)	0.085
Diagnosis, % SCD/MCI/Dementia	57.0/23.0/20.0	44.4/24.7/30.9	0.086
APOE4, %	43.6	36.2	0.314
[1]CSF Aβ42/40 ratio	0.08 (0.03)	0.08 (0.03)	0.815
**Depression**	**N=132**	**N=179**	
Age, mean (SD)	59.0 (6.6)	59.0 (6.6)	0.835
Sex,% female	49.7	66.4	**0.003**
Education, mean (SD)	13.3 (3.1)	13.4 (3.4)	0.471
Diagnosis, % SCD/MCI/Dementia	51.6/20.4/28.0	56.4/25.5/18.1	0.109
APOE4, %	46.9	37.9	0.144
[1]CSF Aβ42/40 ratio	0.08 (0.03)	0.08 (0.02)	**0.045**
**Thyroid disease**	**N=280**	**N=31**	
Age, mean (SD)	58.8 (6.7)	59.0 (6.2)	0.828
Sex, % female	46.2	65.4	**0.001**
Education, mean (SD)	13.2 (3.1)	13.5 (3.3)	0.413
Diagnosis, % SCD/MCI/Dementia	47.7/25.0/27.3	58.1/22.3/19.6	0.155
APOE4, %	43.1	29.0	0.190
[1]CSF Aβ42/40 ratio	0.08 (0.03)	0.08 (0.03)	0.229

Continuous data are shown as mean (SD) and categorical as % frequencies. Between-group differences were calculated by Mann-Whitney U test for continuous variables or chi-square for categorical. $P<0.05$ was considered statistically significant. [1]Data available for 285 participants.

Table S3. Comparisons of mean group differences on AD plasma biomarkers by comorbidities with FDR adjusted values.

Comorbidity	No (mean, SD)	Yes (mean, SD)	P value	P value FDR	[1]P value CSF adj	[1]P value CSF adj FDR
**Cardiovascular**	**N=153**	**N=158**				
Aβ42/40 ratio	0.07 (0.02)	0.07 (0.01)	0.920	0.920	0.628	0.860
Aβ42, ng/L	8.0 (1.9)	8.3 (2.1)	0.178	0.271	0.530	0.860
Aβ40, ng/L	115 (20.6)	123 (23.5)	0.157	0.271	0.243	0.860
p-tau217, ng/L	0.33 (0.56)	0.25 (0.39)	**0.022**	0.097	0.578	0.860
p-tau217/ Aβ42	0.05 (0.09)	0.03 (0.06)	**0.006**	0.050	0.264	0.860
p-tau181, ng/L	9.0 (7.1)	9.5 (6.8)	0.181	0.271	0.552	0.860
p-tau231, ng/L	17.6 (5.2)	18.6 (7.5)	0.257	0.330	0.917	0.917
NfL, ng/L	20.6 (14.9)	21.8 (15.9)	0.529	0.596	0.669	0.860
GFAP, ng/L	118 (85.3)	123 (76.9)	0.161	0.271	0.829	0.917
**Diabetes**	**N=259**	**N=52**				
Aβ42/40 ratio	0.07 (0.02)	0.06 (0.01)	0.475	0.591	**0.013**	0.121
Aβ42, ng/L	8.1 (1.9)	8.3 (2.3)	0.483	0.591	0.902	0.902
Aβ40, ng/L	117 (19.5)	127 (32.5)	0.109	0.475	**0.027**	0.121
p-tau217, ng/L	0.30 (0.47)	0.27 (0.53)	0.211	0.475	0.849	0.902
p-tau217/Aβ42	0.04 (0.07)	0.04 (0.08)	0.147	0.475	0.895	0.902
p-tau181, ng/L	9.2 (7.1)	9.3 (6.3)	0.180	0.475	0.598	0.897
p-tau231, ng/L	17.9 (5.5)	19.2 (10.2)	0.591	0.591	0.176	0.528
NfL, ng/L	20.7 (13.5)	23.6 (21.8)	0.562	0.591	0.370	0.666
GFAP, ng/L	121 (81.5)	120 (79.5)	0.303	0.546	0.262	0.590
**Dyslipidemia**	**N=230**	**N=81**				
Aβ42/40 ratio	0.07 (0.01)	0.07 (0.02)	0.995	0.995	0.855	0.946
Aβ42, ng/L	8.0 (1.9)	8.5 (2.2)	**0.032**	0.143	0.105	0.471

Aβ40, ng/L	116 (21.3)	128 (23.7)	**0.003**	**0.028**	**0.022**	0.196
p-tau217, ng/L	0.29 (0.48)	0.29 (0.48)	0.550	0.946	0.522	0.937
p-tau217/Aβ42	0.04 (0.08)	0.04 (0.07)	0.180	0.539	0.857	0.946
p-tau181, ng/L	9.1 (7.4)	9.6 (5.8)	0.505	0.946	0.946	0.946
p-tau231, ng/L	17.6 (5.4)	19.6 (8.7)	0.630	0.946	0.221	0.663
NfL, ng/L	20.6 (13.6)	23.0 (18.8)	0.899	0.995	0.447	0.937
GFAP, ng/L	119 (82.3)	125 (77.7)	0.877	0.995	0.625	0.937
**Depression**	**N=132**	**N=179**				
Aβ42/40 ratio	0.07 (0.01)	0.07 (0.01)	0.976	0.976	0.632	0.975
Aβ42, ng/L	8.1 (2.1)	8.2 (2.0)	0.943	0.976	0.901	0.975
Aβ40, ng/L	118 (23.4)	120 (21.8)	0.954	0.976	0.720	0.975
p-tau217, ng/L	0.36 (0.53)	0.24 (0.34)	**0.014**	0.129	0.114	0.731
p-tau217/Aβ42	0.05 (0.09)	0.03 (0.05)	**0.039**	0.177	0.220	0.731
p-tau181, ng/L	9.6 (7.6)	9.0 (6.5)	0.302	0.544	0.843	0.975
p-tau231, ng/L	18.9 (7.4)	17.6 (5.7)	0.104	0.235	0.637	0.975
NfL, ng/L	21.3 (15.9)	21.1 (14.6)	0.744	0.976	0.975	0.975
GFAP, ng/L	121 (73.0)	120 (86.7)	0.100	0.235	0.244	0.731
**Thyroid disease**	**N=280**	**N=31**				
Aβ42/40 ratio	0.07 (0.02)	0.07 (0.01)	0.340	0.683	0.520	0.777
Aβ42, ng/L	8.1 (2.0)	8.4 (1.8)	0.961	0.961	0.531	0.777
Aβ40, ng/L	119 (21.8)	124 (20.7)	0.320	0.683	0.188	0.777
p-tau217, ng/L	0.29 (0.49)	0.29 (0.34)	0.435	0.683	0.777	0.777
p-tau217/Aβ42	0.04 (0.08)	0.04 (0.04)	0.531	0.683	0.521	0.777
p-tau181, ng/L	9.01 (6.1)	10.8 (12.4)	0.255	0.683	0.675	0.777
p-tau231, ng/L	18.1 (6.5)	18.9 (6.1)	0.267	0.683	0.608	0.777
NfL, ng/L	21.3 (15.4)	20.5 (13.3)	0.758	0.853	0.304	0.777
GFAP, ng/L	120 (81.4)	124 (79.0)	0.481	0.683	0.757	0.777

Plasma biomarker levels are shown as unadjusted mean (SD). P values are calculated from one-way ANCOVA adjusted for age, sex and education. “P value CSF adj” indicates additional adjustment for CSF Aβ42/40 levels. P<0.05 was considered statistically significant. False discovery rate (FDR) correction (Benjamini–Hochberg, q = 0.05) was applied within each comorbidity factor across the biomarkers. Abbreviations: Aβ, amyloid β; NfL, neurofilament light protein; GFAP, glial fibrillary acidic protein; p-tau, phosphorylated tau.

[1]Data available for 285 participants

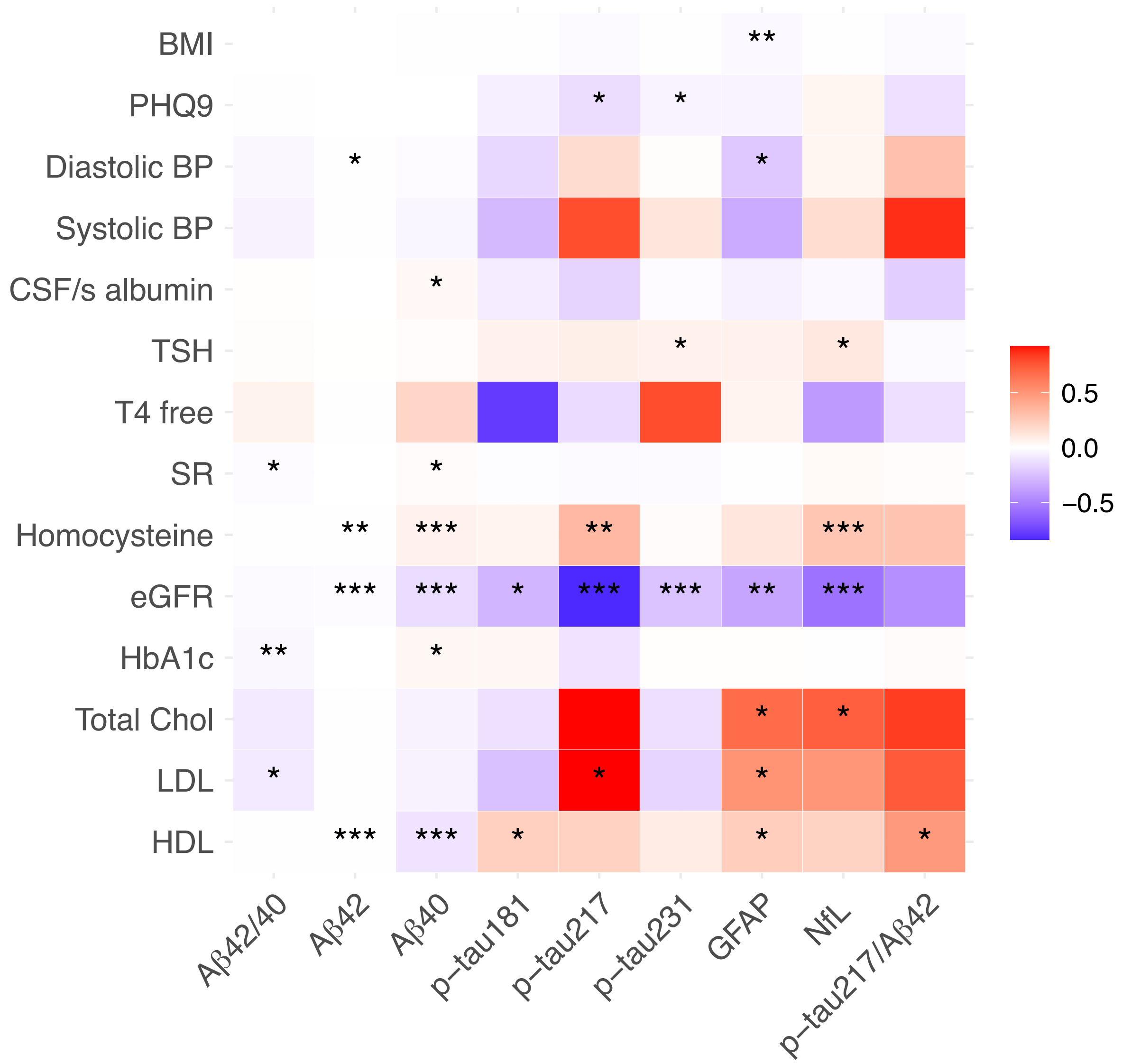

Figure S8. Associations heatmap between comorbidity measures and plasma biomarkers. Standardized beta coefficients from linear regression models adjusted for age, sex, education and diagnosis indicated in red/blue depending on the direction of the association (red: positive association, blue: negative association). Analyses were performed after exclusion of statistical outliers (defined by standardized residuals >±3 or Cook's distance). ns p ≥0.05, * p <0.05, ** p<0.01, *** p<0.001. Abbreviations: Aβ, amyloid β; BMI, body mass index; BP, blood pressure; CSF/S, cerebrospinal fluid/serum; GFAP, glial fibrillary acidic protein; HbA1c, Hemoglobin A1C; HDL, high density lipoprotein; LDL, low density lipoprotein; NfL,

neurofilament light protein; p-tau, phosphorylated tau; SR, sedimentation rate; T4 free, thyroxine; TSH, thyroid stimulating hormone; PHQ-9, patient health questionnaire 9.

Table S4. Associations between plasma biomarkers and comorbidity measures as predictors, with FDR-adjusted p-values.

Predictor	Biomarker	Beta coef	P value	P value FDR	Sig FDR
**HDL**	Aβ40	-0.27	0.001	0.007	**Yes**
**eGFR**	Aβ40	-0.295	0.000007	0.000098	**Yes**
**HDL**	Aβ42	-0.25	0.001	0.007	**Yes**
**Homocysteine**	Aβ42	0.17	0.007	0.0327	**Yes**
**eGFR**	Aβ42	-0.257	0.00011	0.00154	**Yes**
**BMI**	GFAP	-0.21	0.001	0.014	**Yes**
**eGFR**	GFAP	-0.152	0.004	0.028	**Yes**
**Homocysteine**	NFL	0.17	0.001	0.007	**Yes**
**eGFR**	NFL	-0.179	0.000802	0.007	**Yes**
**eGFR**	p-tau231	-0.199	0.001	0.014	**Yes**
**BMI**	Aβ40	-0.04	0.588	0.755	No
**CSF/s albumin**	Aβ40	0.12	0.065	0.228	No
**Diastolic BP**	Aβ40	0.02	0.758	0.816	No
**HbA1c**	Aβ40	0.08	0.213	0.497	No
**Homocysteine**	Aβ40	0.16	0.011	0.0513	No
**LDL**	Aβ40	-0.04	0.528	0.755	No
**PHQ9**	Aβ40	0.03	0.631	0.755	No
**SR**	Aβ40	0.1	0.095	0.266	No
**Systolic BP**	Aβ40	0	0.941	0.941	No
**T4 free**	Aβ40	0.05	0.451	0.755	No
**TSH**	Aβ40	0.05	0.429	0.755	No
**Total Chol**	Aβ40	-0.03	0.647	0.755	No
**BMI**	Aβ42	0	0.966	0.966	No

**CSF/s albumin**	Aβ42	0.08	0.217	0.38	No
**Diastolic BP**	Aβ42	-0.12	0.046	0.161	No
**HbA1c**	Aβ42	0.01	0.904	0.966	No
**LDL**	Aβ42	-0.11	0.088	0.222	No
**PHQ9**	Aβ42	0.04	0.582	0.815	No
**SR**	Aβ42	0.01	0.82	0.957	No
**Systolic BP**	Aβ42	-0.06	0.361	0.562	No
**T4 free**	Aβ42	0.03	0.656	0.835	No
**TSH**	Aβ42	0.1	0.095	0.222	No
**Total Chol**	Aβ42	-0.08	0.199	0.38	No
**BMI**	Aβ42/40	0.01	0.865	0.886	No
**CSF/s albumin**	Aβ42/40	-0.01	0.886	0.886	No
**Diastolic BP**	Aβ42/40	-0.14	0.021	0.294	No
**HDL**	Aβ42/40	-0.01	0.852	0.886	No
**HbA1c**	Aβ42/40	-0.07	0.246	0.574	No
**Homocysteine**	Aβ42/40	0.05	0.41	0.717	No
**LDL**	Aβ42/40	-0.08	0.212	0.574	No
**PHQ9**	Aβ42/40	0.01	0.862	0.886	No
**SR**	Aβ42/40	-0.08	0.181	0.574	No
**Systolic BP**	Aβ42/40	-0.08	0.216	0.574	No
**T4 free**	Aβ42/40	-0.01	0.816	0.886	No
**TSH**	Aβ42/40	0.07	0.223	0.574	No
**Total Chol**	Aβ42/40	-0.05	0.383	0.717	No
**eGFR**	Aβ42/40	-0.04	0.543	0.845	No
**CSF/s albumin**	GFAP	-0.07	0.138	0.215	No
**Diastolic BP**	GFAP	-0.08	0.104	0.215	No
**HDL**	GFAP	0.09	0.083	0.215	No
**HbA1c**	GFAP	-0.07	0.135	0.215	No

**Homocysteine**	GFAP	0.08	0.13	0.215	No
**LDL**	GFAP	0.08	0.086	0.215	No
**PHQ9**	GFAP	0.01	0.808	0.808	No
**SR**	GFAP	-0.04	0.362	0.5	No
**Systolic BP**	GFAP	-0.02	0.646	0.754	No
**T4 free**	GFAP	-0.02	0.747	0.804	No
**TSH**	GFAP	0.04	0.393	0.5	No
**Total Chol**	GFAP	0.11	0.033	0.154	No
**BMI**	NFL	-0.08	0.214	0.428	No
**CSF/s albumin**	NFL	0.04	0.419	0.692	No
**Diastolic BP**	NFL	-0.01	0.905	0.941	No
**HDL**	NFL	0.08	0.116	0.315	No
**HbA1c**	NFL	0	0.941	0.941	No
**LDL**	NFL	0.03	0.538	0.753	No
**PHQ9**	NFL	0.04	0.445	0.692	No
**SR**	NFL	0.08	0.113	0.315	No
**Systolic BP**	NFL	0.03	0.612	0.779	No
**T4 free**	NFL	0	0.917	0.941	No
**TSH**	NFL	0.08	0.109	0.315	No
**Total Chol**	NFL	0.08	0.135	0.315	No
**BMI**	p-tau181	-0.13	0.089	0.208	No
**CSF/s albumin**	p-tau181	-0.1	0.107	0.214	No
**Diastolic BP**	p-tau181	-0.07	0.222	0.388	No
**HDL**	p-tau181	0.14	0.025	0.133	No
**HbA1c**	p-tau181	-0.02	0.758	0.944	No
**Homocysteine**	p-tau181	0.06	0.299	0.465	No
**LDL**	p-tau181	0	0.974	0.974	No
**PHQ9**	p-tau181	-0.12	0.036	0.133	No
**SR**	p-tau181	-0.03	0.551	0.771	No

**Systolic BP**	p-tau181	-0.11	0.077	0.208	No
**T4 free**	p-tau181	-0.01	0.877	0.944	No
**TSH**	p-tau181	0.12	0.038	0.133	No
**Total Chol**	p-tau181	0.01	0.871	0.944	No
**eGFR**	p-tau181	-0.129	0.038	0.133	No
**BMI**	p-tau217	-0.12	0.076	0.213	No
**CSF/s albumin**	p-tau217	-0.13	0.015	0.098	No
**Diastolic BP**	p-tau217	0.03	0.62	0.723	No
**HDL**	p-tau217	0.07	0.187	0.373	No
**HbA1c**	p-tau217	-0.04	0.474	0.664	No
**Homocysteine**	p-tau217	0.12	0.028	0.098	No
**LDL**	p-tau217	0.07	0.172	0.373	No
**PHQ9**	p-tau217	-0.12	0.025	0.098	No
**SR**	p-tau217	0.01	0.905	0.905	No
**Systolic BP**	p-tau217	0.04	0.436	0.664	No
**T4 free**	p-tau217	-0.02	0.738	0.795	No
**TSH**	p-tau217	0.03	0.614	0.723	No
**Total Chol**	p-tau217	0.07	0.213	0.373	No
**eGFR**	p-tau217	0.163	0.004	0.056	No
**BMI**	p-tau217/Aβ42	-0.11	0.099	0.277	No
**CSF/s albumin**	p-tau217/Aβ42	-0.14	0.011	0.14	No
**Diastolic BP**	p-tau217/Aβ42	0.05	0.296	0.452	No
**HDL**	p-tau217/Aβ42	0.13	0.025	0.14	No
**HbA1c**	p-tau217/Aβ42	-0.04	0.504	0.641	No
**Homocysteine**	p-tau217/Aβ42	0.07	0.226	0.396	No
**LDL**	p-tau217/Aβ42	0.09	0.092	0.277	No
**PHQ9**	p-tau217/Aβ42	-0.12	0.03	0.14	No
**SR**	p-tau217/Aβ42	0.01	0.884	0.952	No
**Systolic BP**	p-tau217/Aβ42	0.05	0.323	0.452	No

**T4 free**	p-tau217/Aβ42	-0.02	0.664	0.775	No
**TSH**	p-tau217/Aβ42	0	0.999	0.999	No
**Total Chol**	p-tau217/Aβ42	0.08	0.152	0.355	No
**eGFR**	p-tau217/Aβ42	-0.075	0.182	0.364	No
**BMI**	p-tau231	-0.11	0.113	0.338	No
**CSF/s albumin**	p-tau231	-0.03	0.606	0.771	No
**Diastolic BP**	p-tau231	-0.02	0.75	0.843	No
**HDL**	p-tau231	0.14	0.019	0.133	No
**HbA1c**	p-tau231	-0.01	0.843	0.843	No
**Homocysteine**	p-tau231	0.04	0.474	0.737	No
**LDL**	p-tau231	-0.05	0.358	0.626	No
**PHQ9**	p-tau231	-0.1	0.091	0.338	No
**SR**	p-tau231	-0.08	0.169	0.338	No
**Systolic BP**	p-tau231	-0.04	0.534	0.748	No
**T4 free**	p-tau231	0.08	0.141	0.338	No
**TSH**	p-tau231	0.08	0.15	0.338	No
**Total Chol**	p-tau231	-0.02	0.785	0.843	No

Table S5. Optimal thresholds for A+T+ classification by plasma biomarker in the basic and comorbidity-adjusted models, with corresponding sensitivity and specificity.

Plasma Biomarker	Threshold, Basic model	Sens (%) Basic model	Spec (%) Basic model	Threshold, Adjusted model	Sens (%) Adjusted model	Spec (%) Adjusted model	Δthreshold (Adjusted-Basic)
**Discrimination of A+T+**							
**P-tau217/ Aβ42 ratio**	0.024	90.6	88.0	0.022	94.3	83.9	-0.002
**P-tau217**	0.182 ng/L	92.5	86.5	0.185 ng/L	94.3	86.5	0.003
**GFAP**	106.7 ng/L	89.1	72.7	111.0 ng/L	87.3	75.8	4.35
**P-tau231**	20.6 ng/L	69.1	86.0	22.6 ng/L	50.9	93.3	2.0
**P-tau181**	8.2 ng/L	85.5	70.1	8.6 ng/L	74.5	71.6	0.4
**Aβ42/40 ratio**	0.067	78.2	70.1	0.068	78.2	67.5	0.001
**Aβ42**	7.8 ng/L	72.7	71.1	8.9 ng/L	90.9	44.3	1.1
**NfL**	15.8 ng/L	83.6	50.0	15.2 ng/L	80.0	45.9	-0.6
**Aβ40**	114.5 ng/L	50.9	64.4	111.8 ng/L	40.0	69.6	-2.7
**Aβ42/40, p-tau217, NfL, GFAP**	NA	96.2	88.0	NA	96.2	88.5	NA
**Aβ42/40, p-tau181, NfL, GFAP**	NA	90.9	84.0	NA	92.7	85.1	NA
**Aβ42/40, p-tau231, NfL, GFAP**	NA	89.1	85.5	NA	94.5	82.4	NA

Table S6. Optimal thresholds for MCI/dementia classification by plasma biomarker in the basic and comorbidity-adjusted models, with corresponding sensitivity and specificity.

Plasma Biomarker	Threshold Basic model	Sens (%) Basic model	Spec (%) Basic model	Threshold Adjusted model	Sens (%) Adjusted model	Spec (%) Adjusted model	Δthreshold (adjusted-basic)
**Discrimination of MCI/All cause dementia**							
**P-tau217**	0.197 ng/L	49.2	89.8	0.153 ng/L	55.6	83.7	-0.044
**P-tau217/ Aβ42 ratio**	0.023	52.4	87.8	0.024	52.4	87.1	0.001
**NfL**	15.3 ng/L	73.1	53.7	17.9 ng/L	59.2	70.1	2.6
**GFAP**	120.3 ng/L	47.7	82.3	115.8 ng/L	50.8	80.3	-4.5
**P-tau231**	21.2 ng/L	34.9	89.1	20.0 ng/L	41.1	81.6	-1.2
**P-tau181**	10.4 pg/mL	39.2	89.1	10.8 ng/L	32.3	91.8	0.408
**Aβ42/40 ratio**	0.067	50.8	71.4	0.072	66.9	56.5	0.005
**Aβ42**	8.1 ng/L	60.8	63.3	8.9 ng/L	75.4	46.3	0.818
**Aβ40**	113.0 ng/L	41.5	67.3	112.7 ng/L	38.5	.66.7	-0.3
**Aβ42/40, p-tau217, NfL, GFAP**	NA	64.3	93.2	NA	65.1	90.5	NA
**Aβ42/40, p-tau181, NfL, GFAP**	NA	78.5	78.2	NA	71.5	87.8	NA
**Aβ42/40, p-tau231, NfL, GFAP**	NA	76.0	80.3	NA	72.1	84.4	NA

Table S7. Discrimination performance of plasma biomarker models on AD versus SCD participants

Plasma Biomarker	Basic Model AUC (95% CI)	Comorbidity Adjusted Model AUC (95% CI)	P-Value
**Discrimination between AD and SCD**			
**P-tau217/ Aβ42 ratio**	0.971 (0.944-0.998)	0.970 (0.942-0.999)	0.586
**P-tau217**	0.970 (0.942-0.998)	0.968 (0.936-0.999)	0.523
**GFAP**	0.942 (0.908-0.976)	0.942 (0.907-0.977)	0.942
**P-tau231**	0.928 (0.891-0.965)	0.935 (0.900-0.969)	0.283
**NfL**	0.918 (0.880-0.956)	0.927 (0.891 -0.963)	0.280
**P-tau181**	0.916 (0.877-0.954)	0.918 (0.880-0.956)	0.721
**Aβ42/40 ratio**	0.843 (0.787-0.899)	0.874 (0.822-0.926)	0.085
**Aβ42**	0.831 (0.771-0.891)	0.868 (0.817-0.919)	0.074
**Aβ40**	0.753 (0.671-0.835)	0.797 (0.725-0.868)	0.107
**Aβ42/40, p-tau217, NfL,GFAP**	0.980 (0.963-0.998)	0.983 (0.967-0.999)	0.262
**Aβ42/40, p-tau181, NfL,GFAP**	0.971 (0.950-0.992)	0.975 (0.956-0.994)	0.134
**Aβ42/40, p-tau231, NfL,GFAP**	0.978 (0.960-0.995)	0.981 (0.965-0.996)	0.255

Table S8. Optimal thresholds for AD classification by plasma biomarker in the basic and comorbidity-adjusted models, with corresponding sensitivity and specificity.

Plasma Biomarker	Threshold, Basic model	Sens (%) Basic model	Spec (%) Basic model	Threshold, Adjusted model	Sens (%) Adjusted model	Spec (%) Adjusted model	Δthreshold (Adjusted-Basic)
**Discrimination between SCD and AD**							
**P-tau217/ Aβ42 ratio**	0.040	82.6	95.2	0.026	89.1	88.4	-0.014
**P-tau217**	0.215 ng/L	84.8	91.8	0.198 ng/L	87.0	87.8	-0.017
**GFAP**	131.2 ng/L	67.4	89.1	110.9 ng/L	80.4	80.3	-20.3
**P-tau231**	20.1 ng/L	76.1	83.7	20.1 ng/L	71.7	83.7	0
**P-tau181**	9.9 ng/L	69.6	87.1	9.3 ng/L	71.7	79.6	-0.6
**NfL**	18.6 ng/L	73.9	73.5	17.3 ng/L	78.3	69.4	-1.3
**Aβ42/40 ratio**	0.068	78.3	71.4	0.07	80.4	66.0	0.002
**Aβ42**	7.8 ng/L	71.7	70.7	7.6 ng/L	69.6	71.4	-0.2
**Aβ40**	127.9 ng/L	80.4	32.0	100.7 ng/L	89.1	17.7	-27.2
**Aβ42/40, p-tau217, NfL, GFAP**	NA	97.8	93.2	NA	97.8	94.6	NA
**Aβ42/40, p-tau181, NfL, GFAP**	NA	98.3	89.8	NA	98.7	91.8	NA
**Aβ42/40, p-tau231, NfL, GFAP**	NA	97.8	93.9	NA	97.8	91.8	NA

Table S9. Discrimination performance of plasma biomarker models on MTA abnormality.

Plasma Biomarker	Basic Model AUC (95% CI)	Comorbidity Adjusted Model AUC (95% CI)	P-Value
**Discrimination of MTA abnormality**			
**P-tau217**	0.676 (0.604-0.747)	0.690 (0.620-0.760)	0.330
**NfL**	0.674 (0.603-0.746)	0.682 (0.613-0.752)	0.671
**P-tau231**	0.666 (0.595-0.738)	0.670 (0.600-0.740)	0.805
**P-tau217/ Aβ42 ratio**	0.665 (0.592-0.738)	0.680 (0.609-0.751)	0.254
**GFAP**	0.635 (0.562-0.708)	0.650 (0.579-0.722)	0.428
**P-tau181**	0.631 (0.558-0.705)	0.642 (0.569-0.714)	0.589
**Aβ42**	0.619 (0.545-0.693)	0.640 (0.568-0.713)	0.302
**Aβ42/40 ratio**	0.615 (0.541-0.689)	0.638 (0.566-0.711)	0.260
**Aβ40**	0.615 (0.541-0.689)	0.635 (0.562-0.707)	0.377
**Aβ42/40, p-tau217, NfL,GFAP)**	0.690 (0.620-0.760)	0.709 (0.641-0.777)	0.235
**Aβ42/40, p-tau181, NfL,GFAP)**	0.681 (0.610-0.752)	0.690 (0.621-0.758)	0.636
**Aβ42/40, p-tau231, NfL,GFAP)**	0.692 (0.623-0.762)	0.705 (0.637-0.773)	0.414
